# NMDA receptor modulation of glutamate release in activated neutrophils

**DOI:** 10.1016/j.ebiom.2019.08.004

**Published:** 2019-08-08

**Authors:** Ana Gutierrez del Arroyo, Anna Hadjihambi, Jenifer Sanchez, Egor Turovsky, Vitaly Kasymov, David Cain, Tom D. Nightingale, Simon Lambden, Seth G.N. Grant, Alexander V. Gourine, Gareth L. Ackland

**Affiliations:** a Translational Medicine and Therapeutics, William Harvey Research Institute, Barts and The London School of Medicine and Dentistry, Queen Mary University of London, London EC1M 6BQ, United Kingdom; b Centre for Cardiovascular and Metabolic Neuroscience, Neuroscience, Physiology and Pharmacology, University College London, London WC1E 6BT, United Kingdom; c Institute of Cell Biophysics, Federal Research Center, Pushchino Scientific Center for Biological Research, Russian Academy of Sciences, Russia; d Clinical Physiology, Department of Medicine, University College London, United Kingdom; e Centre for Microvascular Research, William Harvey Research Institute, Barts and The London School of Medicine and Dentistry, Queen Mary University of London, London EC1M 6BQ, United Kingdom; f Centre for Clinical Brain Sciences, University of Edinburgh, Edinburgh EH16 4SB, United Kingdom

**Keywords:** Glutamate, Neutrophils, N-Methylaspartate, Neuroinflammation, Systemic, Inflammation

## Abstract

**Background:**

Neutrophil depletion improves neurologic outcomes in experimental sepsis/brain injury. We hypothesized that neutrophils may exacerbate neuronal injury through the release of neurotoxic quantities of the neurotransmitter glutamate.

**Methods:**

Real-time glutamate release by primary human neutrophils was determined using enzymatic biosensors. Bacterial and direct protein-kinase C (Phorbol 12-myristate 13-acetate; PMA) activation of neutrophils in human whole blood, isolated neutrophils or human cell lines were compared in the presence/absence of N-Methyl-d-aspartic acid receptor (NMDAR) antagonists. Bacterial and direct activation of neutrophils from wild-type and transgenic murine neutrophils deficient in NMDAR-scaffolding proteins were compared using flow cytometry (phagocytosis, reactive oxygen species (ROS) generation) and real-time respirometry (oxygen consumption).

**Findings:**

Both glutamate and the NMDAR co-agonist d-serine are rapidly released by neutrophils in response to bacterial and PMA-induced activation. Pharmacological NMDAR blockade reduced both the autocrine release of glutamate, d-serine and the respiratory burst by activated primary human neutrophils. A highly specific small-molecule inhibitor ZL006 that limits NMDAR-mediated neuronal injury also reduced ROS by activated neutrophils in a murine model of peritonitis, via uncoupling of the NMDAR GluN2B subunit from its' scaffolding protein, postsynaptic density protein-95 (PSD-95). Genetic ablation of PSD-95 reduced ROS production by activated murine neutrophils. Pharmacological blockade of the NMDAR GluN2B subunit reduced primary human neutrophil activation induced by *Pseudomonas fluorescens*, a glutamate-secreting Gram-negative bacillus closely related to pathogens that cause hospital-acquired infections.

**Interpretation:**

These data suggest that release of glutamate by activated neutrophils augments ROS production in an autocrine manner via actions on NMDAR expressed by these cells.

**Fund:**

GLA: Academy Medical Sciences/Health Foundation Clinician Scientist. AVG is a Wellcome Trust Senior Research Fellow.

## Highlights

• Neutrophil depletion improves neurologic outcome after injury and infection.
• Pharmacologic NMDAR blockade reduces rapid autocrine release of glutamate/d-serine from activated neutrophils.
• Genetic ablation/small-molecule inhibition of PSD-95 reduces neutrophil ROS.
• NMDAR blockade reduces human neutrophil activated by glutamate-secreting bacteria.
• Activated neutrophils may exacerbate neuronal injury in various forms of critical illness through the release of glutamate.

## Graphical abstract

**Figure:**
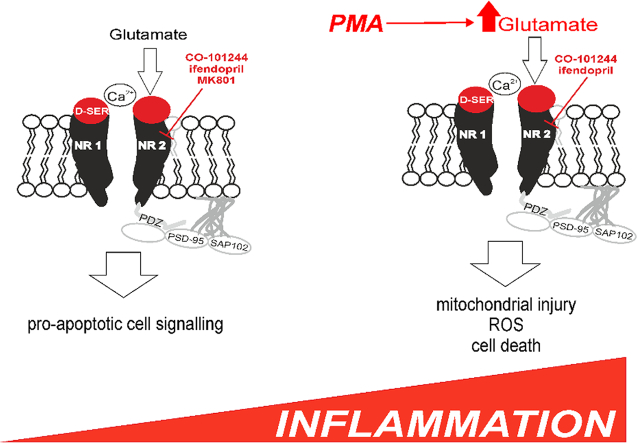

**Research in context**

**Evidence before this study**

Neurologic dysfunction is strongly linked to adverse outcomes in critical illness, yet often unrelated to the inciting pathology. The mechanisms underlying neurologic morbidity in critical illness remain incompletely understood, but systemic inflammation and alterations in *N*-methyl-d-aspartate receptor signalling are common features.

**Added value of this study**

Our data suggest that autocrine release of potentially neurotoxic quantities of glutamate in activated neutrophils augments ROS production.

**Implications of all the available evidence**

NMDAR signalling modulates acute inflammation, contributing to neurologic injury. These findings suggest that neutrophils may contribute to neurologic infection and injury by mimicking glutamate release synonymous with neuronal injury.

Alt-text: Unlabelled Box

## 1 Introduction

Neurologic dysfunction is strongly linked to adverse outcomes and excess mortality in critical illness yet is often unrelated to the inciting pathology [1]. The mechanisms underlying neurologic morbidity in critical illness remain incompletely understood, but systemic inflammation [2] and alterations in N-methyl-d-aspartate receptor (NMDAR) signalling are common features [3,4].

A significant contributor to brain injury is the release of neurotoxic quantities of the neurotransmitter glutamate, [5] which activates intracellular protein kinase-C (PKC) and neuronal NADPH oxidase leading to the injurious production of superoxide [6,7]. NADPH oxidase not only contributes to the generation of superoxide in neuroinflammation and neurodegeneration [8] but also a range of immune cells [9]. Activated human immune cells, including myeloid cells, release glutamate [10] [11,12]. Since innate immunity is directly involved in neuronal deterioration after injury [13] and infection [14,15], immune cells may directly contribute to elevated levels of glutamate. Blood glutamate concentrations of patients with brain injury and/or trauma are higher than those in healthy volunteers [16]. Moreover, depletion of neutrophils after neuronal injury secondary to subarachnoid haemorrhage reduces tissue inflammation and cognitive function in mice, which is associated with changes in neuronal NMDAR subunit composition [17].

Gene expression studies in human neutrophils implicate changes in NMDAR following systemic inflammation triggered by trauma [18]. However, blood levels of glutamate are substantially higher (~60 μM) than levels measured in the brain (0.2–20 μM) [19] and do not activate neutrophils in the absence of danger-associated or pathogen-associated molecular patterns. If glutamate contributes to neutrophil activation via NMDAR, it is therefore likely that PKC activation increases the probability of NMDA channel opening by reducing the voltage-dependent Mg^2+^ block of NMDA-receptor channels, as demonstrated by studies in neurons [20].

Here, we examined whether neutrophils may contribute to the pathobiology of systemic inflammation through the regulated release of neurotransmitters signalling mechanisms harnessed by NMDAR. Our findings suggest that neutrophils and neurons share regulatory mechanisms that provide translational insight into how a variety of clinical interventions play a hitherto unappreciated role in interfering with NMDA modulation of innate immunity in the clinical setting.

## 2 Materials and methods

All experiments were performed in accord with the UK Animals (Scientific Procedures) Act (1986) and ARRIVE guidelines [Supplemental Methods].

### 2.1 Neutrophil samples

#### 2.1.1 Primary human neutrophils

All subjects provided written consent for blood collection, following ethical committee approval. (MREC:11/H0722/3). Neutrophils in whole blood and/or isolated human neutrophils were obtained using Ficoll separation followed by positive selection with CD16 microbeads (Miltenyi Biotec, Bisley, UK) achieving >98.5% purity, as assessed by CD16+ surface staining. SiRNA knockdown targeted against GluN1 and GluN2B in primary human neutrophils was undertaken using electroporation (Amaxa nucleofector II, Lonza). Full siRNAs sequences are provided in Supplementary data.

#### 2.1.2 Human neutrophil-like cell line

HL60 cells obtained from ATCC (CCL-240™) were differentiated into neutrophils by adding 1.25% DMSO for 6 days. GluN1B was knocked down in HL60 cells prior to differentiation, using five short hairpin RNA lentiviral clones expressing the pLKO.1-puromycin vector (MISSION shRNA, Sigma, Poole UK; see Supplementary data for full details). We compared the phenotype of the clone that resulted in the highest knockdown of GluN1B with clones where lentiviral transfection was unsuccessful/minimal. Gene expression was quantified by quantitative PCR performed with SYBR Green Supermix (Roche) on an Eppendorf Realplex system. Gene expression was analyzed using the ΔΔCt method, related to expression in the LN229 neuroblastoma cell line [21] (see Supplementary data for primer details).

#### 2.1.3 Murine neutrophils

C57BL/6 mice were obtained from Charles River UK. PSD-95^−/−^ mice were obtained from the Wellcome Trust Sanger Institute. Female and male mice aged 7–12 weeks were used, with each serving as their own control. Neutrophils were isolated from either bone marrow or peritoneal exudates. Bone marrow derived neutrophils, which have similar respiratory burst and phagocytic characteristics to blood neutrophils [22], were extracted from age and gender-matched mice after cervical dislocation to avoid the potential confounding effects of inhalational anaesthesia on NMDAR activity [10]. Non-viable cells were excluded by 7AAD staining. Neutrophils were identified by Ly6G^+^Ly6C^−^CD11b^hi^ staining. For peritoneal neutrophil harvest, zymosan (1 mg·g^−1^; Sigma, UK) was injected intraperitoneally in C57B/6 wild-type mice of either gender (age 6–8 weeks) prior to lavage with 5 ml ice cold PBS 3 h later. Highly purified neutrophils were obtained by negative selection using a neutrophil isolation kit (MACS Miltenyi Biotec), achieving ≥98% purity.

### 2.2 Measurement of glutamate and d-serine release

The design and operation of enzyme-based glutamate by microelectrode biosensors have been described previously [23]. The glutamate biosensor contains glutamate oxidase, which converts glutamate to α-ketoglutarate, driving production of NH_3_ and H_2_O_2_. The glutamate biosensor amperometrically detects H_2_O_2_ produced within the thin enzymatic layer around the microelectrode tip with a response time of <10 s. d-Serine biosensor currents are generated by d-Amino acid oxidase (DAAO) catalyzing the oxidative deamination of d-serine. We controlled for the release of non-specific electroactive species by placing a null sensor, which lacks enzymes, into a non-adherent heated well (37 °C) containing 1.5 × 10^6^ neutrophils ml^−1^ in PBS. Changes in electrical potential of the biosensors were measured using a potentiostat. Sensors were calibrated before and after every experiment to measure the sensitivity of the biosensors. Changes in extracellular calcium concentration in whole blood were measured using an ionised calcium electrode (ABL625, Radiometer, Copenhagen, Denmark). NMDA (20 μM) and d-serine (5 μM) were also added to human whole blood or isolated neutrophils to assess the impact of acute extracellular increases of these neurotransmitters on ROS, in the presence or absence of the ROS inhibitor diphenyleneiodonium chloride (10 μM).

### 2.3 Confocal and fluorescent calcium imaging

Human primary neutrophils were plated on Cell-Tak coated coverslips prior to incubation with polyclonal antibodies and/or blocking peptide (see Supplemental Digital content). Cultures were imaged (Zeiss LSM 510 or Zeiss 800 laser scanning microscope, with either a 20× (N.A.-0.8) or a 40× (NA 1.3) objective). For fluorescent Ca^2+^ imaging studies, HL60 cells differentiated into the neutrophil-like state were loaded for 30 min at room temperature with 5 μM Fura-2 AM and 0.005% pluronic in HEPES-buffered salt solution (HBSS) containing 156 mM NaCl, 3 mM KCl, 2 mM MgSO_4_, 1.25 mM KH_2_PO_4_, 2 mM CaCl_2_, 10 mM glucose, and 10 mM HEPES (pH 7.35). PMA was added first, followed by NMDA [24]. All the experiments were conducted at 37 °C. Fluorescent imaging was performed using an inverted microscope (I × 71; Olympus, Japan), a high-numerical-aperture oil-immersion objective (60×, 1.65 NA) and a cool-charge-coupled-device camera (Andor, USA). Images were acquired and analyzed using Andor IQ software (Andor, USA). [Ca^2+^]_i_ was monitored in single cells using excitation light provided by a xenon arc lamp, the beam passing through a monochromator sequentially at 340 and 380 nm (Cairn Research, Kent, UK). The Fura-2 fluorescence was not calibrated in terms of [Ca^2+^]_i_ because of the difficulty in deconvoluting the strength of the signal from the amount of dye taken up by each cell. The Fura-2 ratio is defined as the ratio of fluorescence emission upon excitation at 340 nm (Ca^2+^-bound form) to emission upon excitation at 380 nm (unbound fluorophore).

### 2.4 Flow cytometry

Neutrophils were identified using forward and side scatter characteristics (Supplementary Figs. 1–3), in combination with specific cell surface antigen for CD16 (clone VEP13), CD14 (clone TUK4) and CD11b (clone M1/70.15.11.5) or the appropriate isotype control (all Miltenyi Biotec). Intracellular staining was performed using BD Cytofix/Cytoperm™ Fixation/Permeabilization Kit (BD Biosciences, Oxford, UK). Serine racemase (clone 7E8) antibody was acquired from Abcam (Cambridge, UK). NMDAR subunits and associated scaffolding proteins were quantified with appropriate secondary antibodies or isotypes as controls, using validated antibodies as indicated: NMDAε1 antibody (clone H-54 rabbit polyclonal IgG); sc-9056, Santa Cruz [25]; NMDAζ1 Antibody (clone C-20; goat polyclonal IgG), sc-1467 (conjugated with phycoerythrin), Santa Cruz [26]; GluN2B-FITC (IgG linear peptide corresponding to mouse NR2B; FCABS332F, Merck Millipore); PSD-95 antibody (clone H-40, rabbit polyclonal IgG, sc-28,941; Santa Cruz) [27]; SAP102 (NE-dlg, 7H11; sc-134,400, Santa Cruz). Flow cytometry dot plots were based on comparison with isotype controls, fluorescence minus one (FMO), permeabilized and unpermeabilized unstained cells. Acquired data (Cyan ADP (Beckman Coulter, MI USA); FACSCalibur) were analyzed using Kaluza (Beckman Coulter, MI USA) and/or CellQuest (BD Biosciences, Oxford, UK) software.

### 2.5 Neutrophil function

Three separate experimental models were employed: (i) highly purified human neutrophils, (ii) human whole blood and (iii) murine neutrophils recruited in response to zymosan peritonitis.

#### 2.5.1 Neutrophil oxidative burst

Opsonized *Escherichia coli* (3 × 10^6^ bacteria), fMLP (5 μM), PMA (0.9 μM) or wash solution (negative control) were added to blood or isolated neutrophils for 10 min at 37 °C in a water bath. Respiratory burst activity was assessed using dihydrorhodamine-123 (Phagoburst®, Orpegen, Germany). After paraformaldehyde fixation, quantification of ROS was estimated by median fluorescence intensity within the CD16^+^ neutrophil population.

#### 2.5.2 Neutrophil oxygen consumption

We used real-time respirometry (Seahorse XF, North Billerica, MA, USA) to quantify oxygen consumption in human, murine and HL60 cells (0.2–1 × 10^6^ cells/well). Co 101244 (25–250 μM) or medium were injected, 10 min prior to injection of vehicle control (DMSO) or PMA (500ɳM; Sigma-Aldrich).

#### 2.5.3 Neutrophil phagocytosis

200 μl heparinized whole peripheral blood was incubated with 40 μl opsonized FITC-labeled *Escherichia coli* for 10 min at 37 °C in a water bath. Samples remaining on ice served as negative controls. To stop phagocytosis, 200 μl Phagotest® quenching solution was added to each sample at the end of the incubation time. Samples were then washed with 3 ml Phagotest® washing solution twice and lysed with 2 ml Phagotest® lysis solution for 20 min at room temperature. PI excluded dead/non-viable cells. After a further wash, neutrophils were either processed immediately or stained with anti-CD16–APC for 20 min at 4 °C, before the final assay step. Neutrophil subsets were defined according to their scatter properties and CD16+ expression. Experiments with a minimum n = 4, performed in 3 separate experiments.

### 2.6 Bacterial culture

The glutamate-producing bacillus *Pseudomonas fluorescens* proliferates in mouse brain homogenates at 4 °C.(Tatara et al., 2008) We incubated *P. fluorescens* (10^6^ cfus·ml^−1^) with 3 × 10^6^ highly purified (97 ± 1%) primary human neutrophils (10^6^·ml^−1^) obtained from healthy volunteers (n = 4) for 18 h in Dulbecco's Modified Eagle's medium (without antibiotics). Co 101244 (10–1000 μM) or vehicle control were added to these samples. This strain was resistant to penicillin, cephalosporins and clindamycin (Royal Veterinary College Diagnostic Laboratories, North Mymms, Herts UK).

### 2.7 Immunoblots

Membranes were incubated with the following primary antibodies (from Santa Cruz Biotechnology, Heidelberg, Germany, unless otherwise stated): GluN1 (sc-9056), NE-dlg (SAP102; sc-134,400), PSD-95 (sc-28,941), ERK (1:1000; Cell Signaling Technology, Leiden; #4695), phospho-ERK (1:1000; Cell Signaling Technology, Leiden; #4370), AKT (1:1000; Cell Signaling Technology, Leiden; #9272), phospho-AKT (S473) (1:1000; Cell Signaling Technology, Leiden; #9271), NOX-2 (1:1000; Abcam, Oxford; ab129068). Loading control used was GAPDH (1:1000; Cell signaling). Secondary antibodies (1:2000; Dako, Stockport, UK or Cell Signaling Technology, Leiden) were rabbit anti mouse HRP or goat anti-rabbit HRP, as indicated. Membranes were developed using ECLTM reagent and Hyperfilm ECL (Amersham, UK).

### 2.8 Replicates

Each experiment was performed at least 3 times, with ≥3 independent biological data points obtained.

### 2.9 Statistics

#### 2.9.1 Randomisation and blinding

All samples/mice were randomised to receive specified interventions, using Research Randomizer [28]. Analyzers were blinded to the identify of treatments/genotypes.

#### 2.9.2 Statistical testing

Data were processed by analysers blinded to group assignments. For experiments with values measured over time, 2-way ANOVA analysis was used. Student's *t*-tests was used for normally distributed data, having applied the Kolmogorov-Smirnov normality test. P values < 0.05 were considered significant.

#### 2.9.3 Sample size estimation

For paired samples, to detect a mean difference of 30% (±20 SD) in dihydrorhodamine fluorescence, ≥7 samples per group would be required (α = 0.05; 1-β = 0.9; two-tailed). For unpaired samples, to detect a mean difference of 20 percentage points (±10 SD) in dihydrorhodamine fluorescence, ≥6 samples per group would be required (α = 0.05; 1-β = 0.9; two-tailed). Sample size and statistical analyses were performed using NCSS 11 software (Kaysville, Utah, USA).

## 3 Results

### 3.1 Release of glutamate by activated neutrophils

**Fig. 1:**
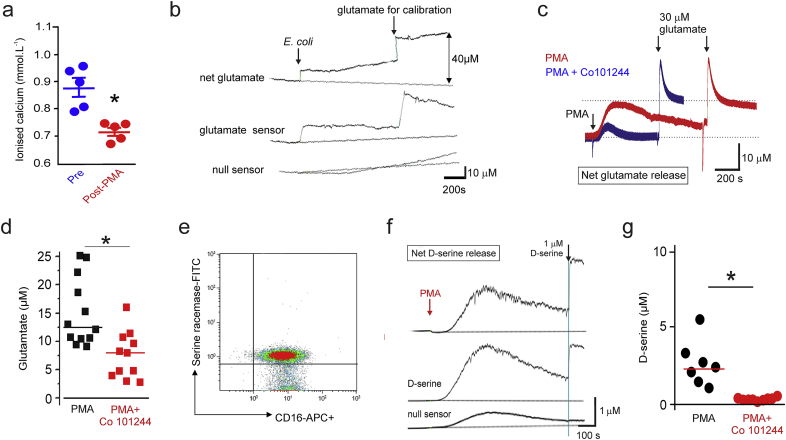
Release of glutamate and d-serine by activated human neutrophils. A) Ionised calcium falls in whole human blood after PMA stimulation; calcium levels are shown from the same blood samples before and after PMA (*p < 0.01, paired *t*-test, n = 5 separate samples). B) Real-time recordings of glutamate release (negative deflection) by isolated primary human neutrophils before and after application of *Escherichia coli* in whole blood. Glutamate biosensor was calibrated with 40 μM glutamate at the end of the experiment. The null sensor, which lacks enzymes, detects release of non-specific electroactive species. The difference between glutamate and null sensor readings quantifies glutamate. C) Real-time recordings of glutamate release by isolated primary human neutrophils before and after application of PMA. PMA-induced glutamate release was reduced in the presence of Co 101244 (100 μM). Glutamate biosensors were calibrated with 30 μM glutamate at the end of the experiment. The null sensor detects non-specific interferents. D) Summary data for glutamate release from isolated primary human neutrophils following PMA-activation in the presence/absence of Co 101244 (100 μM; n = 10 separate samples; * p < 0.05, by paired *t*-test). E) Density plot showing that serine racemase is present on cell surface of CD16+ primary human neutrophils. Superimposed lines show FMO/isotype control defined boundaries. F) Real-time recording of d-serine release by isolated primary human neutrophils before and after application of PMA. d-serine biosensors were calibrated with 1 μM d-serine at the end of the experiment. G) Summary data for d-serine release from isolated primary human neutrophils following PMA-activation in the presence/absence of Co 101244 (100 μM; n = 10 separate samples; * p < 0.05, paired *t*-test).

In whole blood, a rapid decline in ionised calcium after PMA was observed, indicating that high calcium permeability may contribute to primary neutrophil activation (Fig. 1A). Glutamate excess damages neurons through facilitating calcium influx and neuronal release of superoxide by activating neuronal NADPH oxidase [7]. This process is predominantly mediated by GluN2B NMDAR subunit-scaffolding protein interactions [29]. We confirmed GluN2B subunit transcription and protein expression in both primary neutrophils and HL60 neutrophil-like cells (Supplementary data). Stimulation of either isolated human neutrophils or heparinized whole blood with either opsonised *Escherichia coli* (Fig. 1B), a common Gram-negative pathogen in meningitis [30], or PMA (Fig. 1C) induced rapid release of glutamate in micromolar quantities (Fig. 1D). Given the rapid, substantial rise in glutamate after neutrophil activation and accompanying decline in calcium in whole blood, we next tested whether GluN2B blockade may reduce glutamate release. We found that glutamate release was reduced in the presence of the specific GluN2B antagonist Co 101244 (Fig. 1C,D).

In neurons, d-serine is a key determinant of glutamate neurotoxicity, acting at the NMDAR as a co-agonist [31]. Serine racemase, the enzyme which converts l-serine to d-serine, [32] was present on the surface of human neutrophils (Fig. 1E). Accordingly, we also detected significant release of d-serine following opsonised *Escherichia coli* (Supplementary data) or PMA stimulation of primary human neutrophils (Fig. 1F). As we found with glutamate, d-serine release was also reduced in the presence of the specific GluN2B antagonist Co 101244 (Fig. 1G).

Direct application of NMDA and d-serine (at similar concentrations to those we found released following neutrophil activation) caused rapid loss of surface CD16^+^ in isolated primary human neutrophils (Supplementary Fig. 4), indicative of apoptosis [33]. By contrast, there was minimal change in surface CD11b expression, which increases dramatically when neutrophils are activated [34] or reactive oxygen species either in the presence or absence of the ROS inhibitor diphenyleneiodonium chloride (Supplementary Fig. 4).

### 3.2 GluN2B NMDAR subunit augments neutrophil ROS production

In neurons, high levels of glutamate cause tissue injury by calcium influx through the GluN2B NMDAR subunit, which results in neuronal NADPH oxidase generating ROS [7]. Since neutrophil depletion exacerbates neuronal injury in subarachnoid haemorrhage, we next explored whether selective blockade of the NMDAR GluN2B similarly reduces ROS generation in neutrophils using two separate experimental models.

**Fig. 2:**
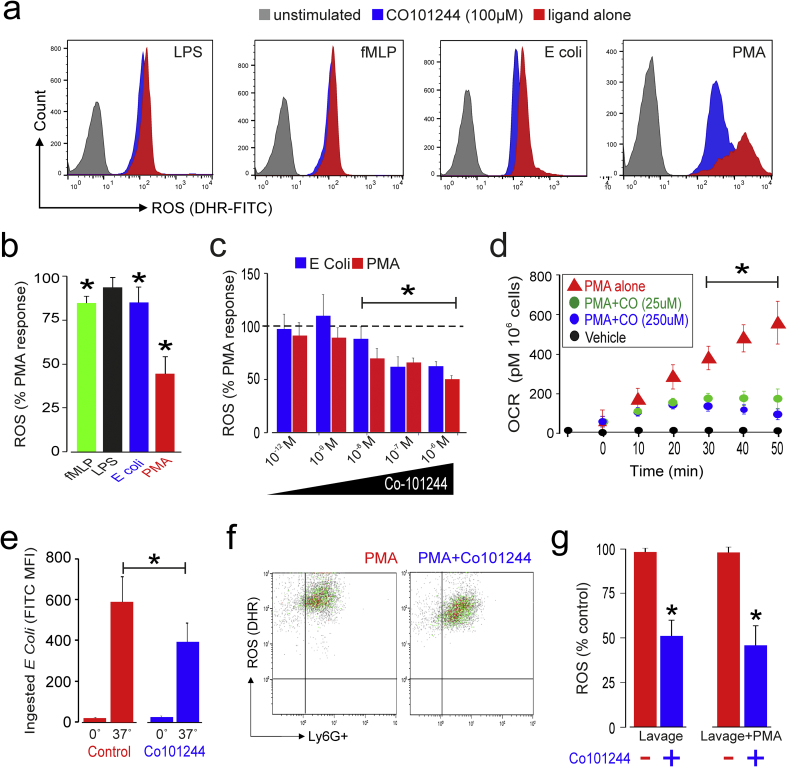
GluN2B NMDAR subunit modulates neutrophil ROS production. (A) Histograms illustrating effect of GluN2B receptor antagonist Co 101244 (100 μM) on ROS generation (quantified by 123-dihydrorhodamine) triggered by lipopolysaccharide (100 ng·ml^−1^), fMLP, and opsonised *E coli* (3 × 10^6^·ml^−1^) in whole blood preparation and direct PKC stimulation using PMA. (B) Population data for GLuN2B antagonist Co 101244 on ROS generation (quantified by 123-dihydrorhodamine) in primary human neutrophils after ligands shown in panel A, presented as %PMA response (i.e. PMA alone with no GluN2B antagonist present; *p < 0.05, ANOVA; n = 5–8). (C) Co 101244 in submicromolar concentrations reduces ROS release (quantified by 123-dihydrorhodamine) from CD16+ neutrophils in whole blood samples following *E Coli* and PMA, standardized as % of PMA response (i.e. PMA alone with no GluN2B antagonist present; mean ± sem, n = 3–7/group; *p < 0.01, ANOVA). (D) Oxygen consumption (measured by direct respirometry (Seahorse XF96)) before, and following, PMA-stimulation in isolated highly purified (>98%) primary human neutrophils pretreated with Co 101244 (25–250 μM) or phosphate-buffered saline control (mean ± sem, *p < 0.05; n = 5, one-way ANOVA, (drug × time)). (E) Primary human neutrophil phagocytosis of opsonized FITC-labeled *E coli* is reduced by GluN2B antagonist Co 101244 (100 μM; *p < 0.05; n = 5 patient samples, paired *t*-test). (F) ROS generation in microbead purified peritoneal neutrophils (Ly6G+) obtained 3 h after intraperitoneal injection of 1 g/g zymosan in C57B/6 mice. GluN2B antagonist Co 101244 was incubated with isolated cells for 30 minutes ex-vivo in presence/absence of PMA. (G) Graph shows summary data for peritoneal neutrophil ROS (quantified by 123-dihydrorhodamine) expressed as %PMA response (i.e. PMA alone with no GluN2B antagonist present) after IP zymosan alone and PMA post-peritoneal harvest. Co 101244 reduced ROS in lavage neutrophils and after stimulation with PMA (p < 0.01, paired *t*-Test; n = 10 mice; mean ± sem).

First, in isolated human neutrophils (purity > 98%), pharmacological blockade of the GluN2B subunit using Co 101244 reduced ROS generation following stimulation with the neutrophil-stimulating bacterial peptide N-formyl Methionyl-Leucyl-Phenylalanine (fMLP), opsonised *E. coli* and the direct protein kinase C agonist PMA (Fig. 2A, B). Consistent with flow cytometry-based measurements of ROS generation (Fig. 2C), pharmacologic blockade of GluN2B also reduced cellular oxygen consumption (the most accurate physiologic measure of neutrophil activation) by PMA-stimulated purified primary human neutrophils (Fig. 2D). In keeping with the observation that genetic NADPH oxidase deficiency results in a decreased ability of neutrophils to phagocytose opsonised bacteria [35], selective GluN2B blockade reduced bacterial phagocytosis in primary human neutrophils (Fig. 2E).

Second, in a murine model, we explored how NMDAR may modulate zymosan-induced peritoneal inflammation, which is characterized at an early stage (4–6 h) by infiltration of neutrophils (Fig. 2F). We found that inhibition of the NMDAR GluN2B reduced ROS generation by Ly6G^+^ peritoneal neutrophils elicited by intraperitoneal zymosan. Pharmacologic inhibition of the NMDAR GluN2B subunit further suppressed ROS generation elicited by PMA applied ex-vivo after intraperitoneal zymosan (Fig. 2G).

### 3.3 NMDAR (GluN2B) expression correlates with maturation of human neutrophils

**Fig. 3:**
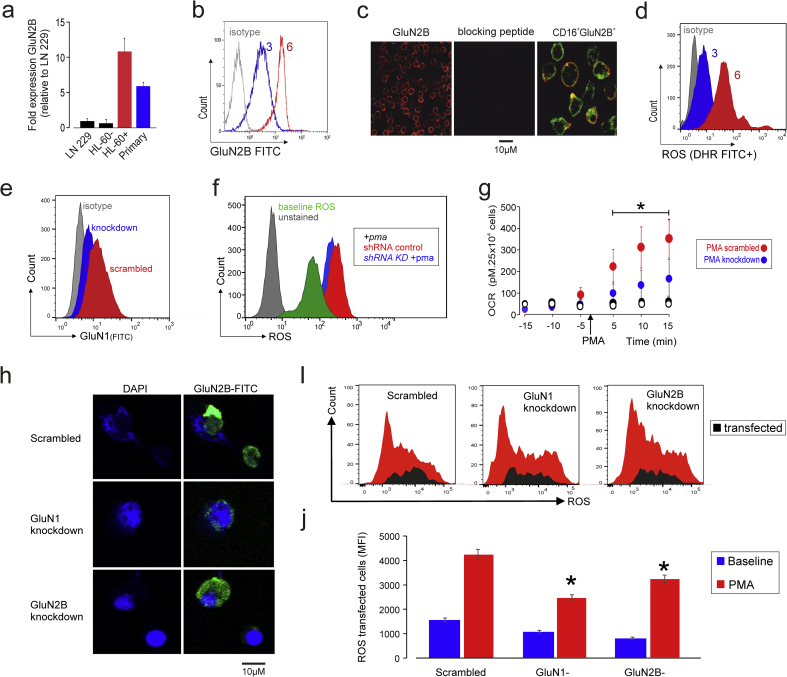
Maturation of GluN2B NMDAR subunit expression in primary human granulocytes and neutrophil-like HL60 cells. (A) RT-PCR showing GluN2B mRNA in primary human neutrophils, undifferentiated (designated as HL60-) and HL60 cells differentiated with DMSO for 6 days (designated as HL60+), referenced to human neuroblastoma LN229 cell line (which expresses NMDAR in low quantities). GluN2B mRNA was higher in DMSO-differentiated cells (p < 0.01; n = 5; mean ± sem) (B) GluN2B expression in differentiated neutrophil-like HL60 cells following 3 days (blue line) or 6 (red line) days treatment with 1.25% DMSO (dimethyl sulfoxide). Isotype control is shown in grey for both timepoints. Numbers above histograms indicate days of DMSO treatment. (C) Confocal images (Zeiss LSM 510) showing cell surface staining for GluN2B, in absence or presence of specific blocking peptide anti- GluN2B Receptor (extracellular), conjugated to ATTO-594 (1:500; Alomone antibodies, Israel). Lower panel shows extracellular co-staining of GluN2B and CD16. (D) Differentiation of HL60 cells with 1.25% DMSO into neutrophil-like cells for up to 6 days is associated with higher increase in ROS (quantified by 123-dihydrorhodamine) following PMA stimulation, compared to HL60 cells differentiated for 3 days with 1.25% DMSO. n = 3–5 group; 3 independent experiments. (E) shRNA knockdown of GluN1 in HL60-neutrophil like cell line. (F) Effect of shRNA knockdown of GluN1 in HL60-neutrophil like cell line PMA-induced ROS production, as quantified by 123-dihydrorhodamine (*p < 0.05; n = 3 independent experiments). (G) Oxygen consumption (measured by direct respirometry (Seahorse XF96)) following PMA-stimulation in scrambled (red circles) or GluN1 deficient (blue circles) HL60 cells (mean ± sem, *p < 0.01; n = 5, ANOVA). White and black circles represent unstimulated control cells for each scrambled or GluN1 deficient genotype, respectively. (H) Confocal microscopy expression of GluN2B 24 h after transfection of primary human neutrophils with either scrambled siRNA, or siRNA targeted at knocking down GluN1 or GluN2B NMDAR subunits. (I) Representative histograms of ROS production (quantified by 123-dihydrorhodamine) following PMA activation of primary human neutrophils 24 h after transfection. Black areas indicate Alexa-647 transfected cells (mean (SD)) 22 ± 3% neutrophils were transfected, as determined by flow cytometric analysis of Alexa-647 conjugated to siRNA. (J) Summary ROS data for siRNA knockdown experiments in human primary cells, before (baseline) and after PMA stimulation (mean ± sem, *p < 0.05; n = 3, comparing ROS generation in scrambled versus GluN1 or GluN2B siRNA after PMA stimulation; ANOVA).

Since mature neutrophils release more ROS, we examined whether GluN2B expression changed when HL60 cells are differentiated into neutrophil-like cells. Undifferentiated HL60 cells showed low levels of GluN2B subunit expression, similar to the low quantities known to be expressed in the human neuroblastoma LN229 cell line ([21]; Fig. 3A). An increase in GluN2B subunit transcription (Fig. 3A) and protein expression (Fig. 3B) was observed following differentiation into a neutrophil-like phenotype (Collins et al., 1979). GluN2B was also expressed in highly purified primary CD16+ neutrophils (Fig. 3C). GluN2B subunit upregulation was associated with higher ROS production in response to PMA (Fig. 3D). We next assessed whether genetic reduction in GluN2B expression altered ROS production, by using a short hairpin RNA to reduce the obligatory GluN1 subunit that is required for GluN2B expression [36], by ~50% (Fig. 3E). It should be noted that the HL60 cell line has higher constitutive ROS production, in contrast to primary neutrophils (as demonstrated by baseline ROS quantification; Fig. 3F). Nevertheless, GluN2B subunit upregulation was associated with higher ROS production in response to PMA (Fig. 3F). In differentiated HL60-neutrophil like cells. GluN1 deficient was associated with a smaller increase in oxygen consumption after PMA stimulation (Fig. 3G). siRNA targeted at knocking down GluN1 or GluN2B in primary human neutrophils (Fig. 3H, I) also reduced ROS after PMA stimulation (Fig. 3J).

### 3.4 PKC activation facilitates NMDAR modulation of neutrophil function by calcium influx

**Fig. 4:**
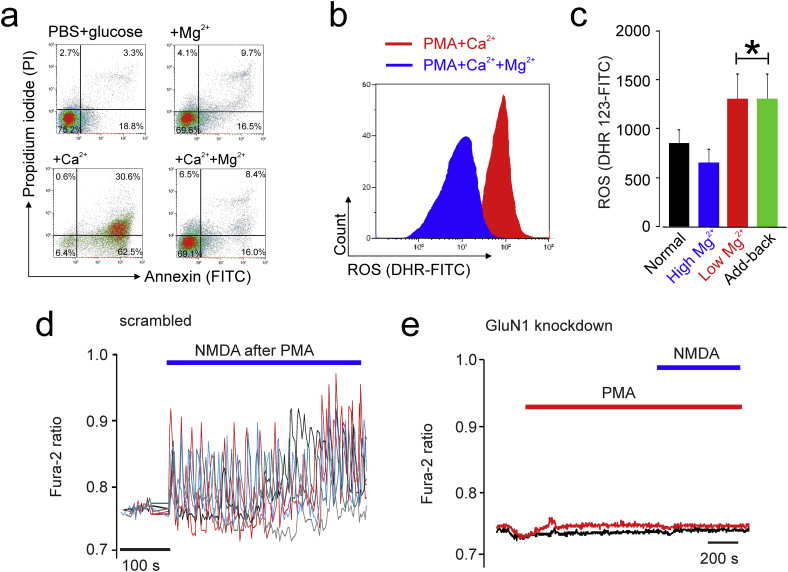
Modulation of NMDAR gating. (A) Apoptosis in isolated primary human neutrophils is augmented when extracellular magnesium is absent. Extracellular calcium was maintained within normal limits. (mean (SE); n = 6 individuals' samples). Representative density plots of annexin V and PI expression shown from 3 independent experiments. (B) PMA-induced neutrophil ROS in human whole blood was augmented following extracellular magnesium depletion using EDTA. Plasma calcium was maintained within normal limits by supplementing the blood with calcium chloride. For clarity, unstained unstimulated control omitted. ROS was quantified by 123-dihydrorhodamine. (C) PMA-induced neutrophil ROS (quantified by 123-dihydrorhodamine) in human whole blood remained unaffected by delayed add-back of magnesium to restore normal plasma levels (≥0.8 mmol L^−1^; mean (SE); n = 10 individuals/group, p < 0.01, ANOVA). Plasma calcium was maintained within normal limits. (D) Calcium oscillations in HL60 neutrophil-like cells triggered by PMA and NMDA (500 μM). Colour traces depict changes in intracellular [Ca^2+^] in five individual HL60 cells over time, representative of 3 separate experiments. (E) Neither PMA nor NMDA were able to trigger Ca^2+^ responses in HL60 mutant cell line where the GluN1 gene was knocked down by shRNA.

We next further examined why circulating neutrophils do not appear to mount a respiratory burst in the presence of high concentrations of blood glutamate alone. Phosphorylation of protein kinase C was not affected by pharmacologic GluN2B inhibition in primary human neutrophils, as assessed by immunoblot and flow cytometry (Supplementary Fig. 5). We examined whether this may be explained by a critical feature of NMDAR neuronal biology, which requires PKC activation to increase the probability of NMDA channel opening by reducing voltage-dependent Mg^2+^ block of NMDA-receptor channels [20]. In isolated primary neutrophils, Mg^2+^ reduced neutrophil apoptosis in the presence of extracellular calcium (Fig. 4A). In whole blood, extracellular magnesium was depleted using EDTA. PMA-induced ROS in primary human neutrophils was augmented in the following depletion of magnesium, with plasma calcium maintained within normal limits by adding back calcium chloride (Fig. 4B). PMA-induced neutrophil ROS in human whole blood remained unaffected by delayed add-back of magnesium to restore normal plasma levels (≥0.8 mmol L^−1^). Using HL60 neutrophil-like cells, we found that NMDA application only after PMA treatment induced Ca^2+^ oscillations (Fig. 4D). Neither PMA nor NMDA were able to trigger Ca^2+^ responses in a HL60 mutant cell line where the GluN1 gene was knocked down by shRNA (Fig. 4D).

### 3.5 NMDAR scaffolding protein interaction with nNOS mediates ROS generation by human neutrophils

**Fig. 5:**
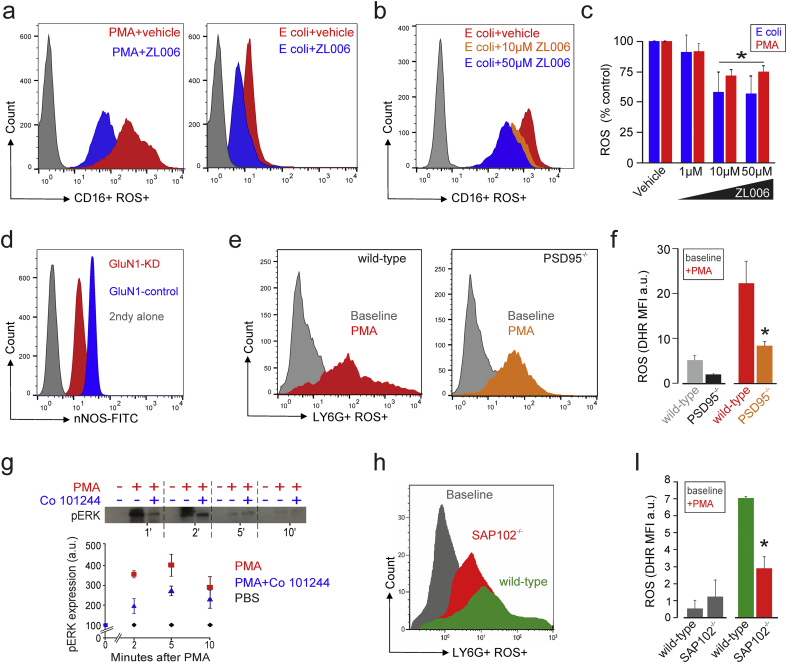
GluN2B-MDAR scaffolding proteins modulate neutrophil ROS generation. (A) Histograms illustrating ROS generation (quantified by 123-dihydrorhodamine) following stimulation with *E. coli* and PMA-which is reduced by ZL006 (1–50 μM) in purified primary human neutrophils (n = 7). (B) *E. coli*-induced ROS in whole blood samples is reduced by ZL006 in a dose-dependent manner (10–50 μM; n = 7). (C) Summary data showing effect of ZL006 on PMA-induced ROS generation in neutrophils, as quantified by 123-dihydrorhodamine. Data expressed as % respective control (mean ± sem; n = 7 subjects; *p = 0.01, ANOVA). (D) shRNA knockdown of GluN1B is associated with reduced nNOS protein expression in differentiated HL60 neutrophil-like cells. (E) PMA-induced ROS production (quantified by 123-dihydrorhodamine) is attenuated in bone-marrow derived neutrophils from wild-type and homozygous PSD-95 deficient. (F) Population data for ROS in wild-type and homozygous PSD-95 deficient bone marrow derived neutrophils (mean ± sem; n = 6–7 mice/group; *p = 0.02 unpaired *t*-test). (G) ERK phosphorylation in primary human neutrophils is reduced by GluN2B antagonist Co 101244 following PMA-stimulation phospho-flow cytometry in CD16+ cells, human whole blood (mean ± sem; n = 5 experiments; *p < 0.05; two-way (drug × time) ANOVA with Tukey posthoc comparison). Immunoblot above graph shows similar results obtained in five separate experiments. Numbers below123-dihydrorhodamine immunoblot denote minutes after PMA stimulation (in the presence/absence of GluN2B subunit antagonist Co 101244) or phosphate-buffered saline control. (H) PMA-induced ROS production is attenuated in bone-marrow derived neutrophils from wild-type and homozygous SAP102 deficient mice. (I) Population data for wild-type and homozygous SAP-102 deficient bone marrow derived neutrophils (mean ± sem; n = 6–7 mice/group; *p = 0.04 unpaired *t*-test).

NMDA receptor subunits (GluN1, GluN2A-D) form tetramers that assemble via the cytoplasmic domain of GluN2B with Membrane Associated Guanylate Kinase (MAGUK) scaffold proteins (PSD-95/Dlg4, SAP102/Dlg3, PSD93/Dlg2) to form super-complexes linked to other receptors and signalling proteins [37]. In the absence of the scaffolding protein PSD-95, neuronal injury is reduced [38]. PSD-95 binds both NMDARs and nNOS at excitatory synapses by assembling them into macromolecular signalling complexes. Activation of nNOS requires PSD-95 following NMDAR-mediated excitotoxic calcium influx [39]. The small-molecular inhibitor ZL006 selectively and potently blocks the interaction of nNOS and PSD-95, through which focal cerebral ischemic damage is reduced in animal models of stroke [29]. We therefore tested whether ZL006 also reduced neutrophil ROS generation; we found a similar effect to that described in neurons in both primary human neutrophils (Fig. 5A) and whole blood samples challenged with opsonised *Escherichia coli* (Fig. 5B). These data suggest a similar role for the NMDAR scaffolding protein PSD-95-nNOS complex in neutrophils and neurons (Fig. 5C). Consistent with these data, knockdown of GluN1 in neutrophil-like HL60 cells was associated with a reduction in nNOS protein expression (Fig. 5D).

To further explore a potential role for PSD-95, we also tested to see if PSD-95 genetic deficiency altered the neutrophil respiratory burst. Bone marrow-derived neutrophils from PSD-95^−/−^ mice (Fig. 5E) generated less ROS following PMA challenge, as compared to neutrophils isolated from wild type mice (Fig. 5F). In the central nervous system, NMDAR activation also triggers phosphorylation of ERK- a process involving SAP102 scaffolding protein [40]. We found that blockade of GluN2B inhibits ERK phosphorylation in primary human neutrophils (Fig. 5G). Furthermore, bone marrow-derived neutrophils obtained from SAP102 deficient mice displayed reduced (40)ROS production in response to PMA challenge (Fig. 5H), as compared to neutrophils isolated from wild type mice (p < 0.05; n = 6/group; Fig. 5I).

### 3.6 Regulation of neutrophil apoptosis by NMDAR-mediated signalling

**Fig. 6:**
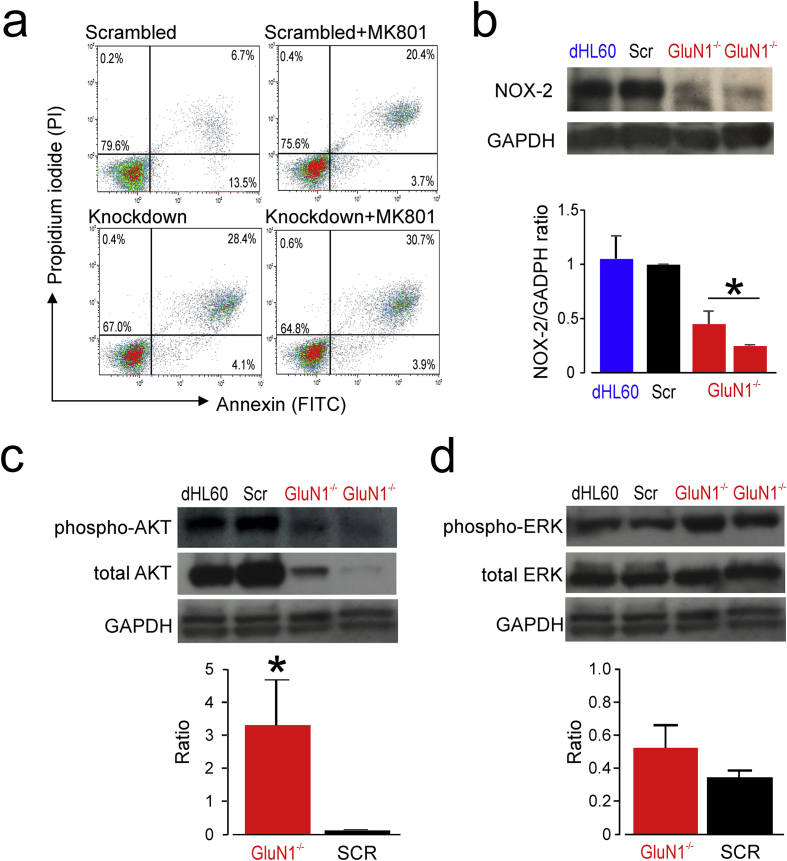
NMDAR modulation of neutrophil survival. (A) Annexin V versus propidium density plots to quantify constitutive apoptosis in HL60-neutrophil like cell line, comparing control (scrambled shRNA) with shRNA knockdown of GluN1 (representative plots of n = 3 experiments). Application of MK801, a non-competitive NMDAR blocker, failed to reduce ROS in cells where GluN1 expression was reduced. In contrast, MK801 increased baseline ROS in scrambled cells. (B) Immunoblot for NADPH oxidase (isoform-2; 1:1000; Cell Signalling) in differentiated, scrambled or GluN1deficient HL60-neutrophil like cell line. GAPDH served as loading control. Densitometry ratios summarised in graph; n = 3; * p = 0.02, by Students *t*-test. (C) Immunoblot for AKT and phosphorylation of AKT (1:1000;Cell Signalling) in differentiated, scrambled or GluN1deficient HL60-neutrophil like cell line. GAPDH served as loading control (Densitometry ratios summarised in graph; n = 3; * p = 0.04, by Students *t*-Test). (D) Immunoblot for ERK and phosphorylation of ERK (1:1000; Cell Signalling) in differentiated, scrambled or GluN1deficient HL60-neutrophil like cell line. Total GAPDH (cytoplasmic and nuclear) served as loading control, hence dual band. (Densitometry ratios are summarised in graph; n = 3).

We next explored whether NMDAR-mediated mechanisms may contribute to the viability of neutrophils, since neuronal viability is controlled, in part, by NMDAR-mediated regulation of phosphorylation of pro-survival kinases [41,42]. In contrast to NMDAR-mediated neuronal injury triggered by sustained elevations in extracellular glutamate, activation of neuroprotective signalling cascades requires tonic, transient exposure of NMDAR to lower extracellular glutamate concentrations [41,42]. Synaptic NMDAR-mediated signalling by glutamate is coupled to the transcriptional control of the glutathione system [43], thereby preventing apoptosis and maintaining neuronal activity [44]. Cell viability was assessed in differentiated neutrophil-like HL60 cells where shRNA transfections reduced NMDAR expression. Application of MK801, a non-competitive NMDAR blocker [45], did not reduce apoptosis in cells when GluN1 expression was reduced (Fig. 6A). In HL60 cells where NMDAR expression was knocked down, the baseline rate of apoptosis was enhanced and MK801 had no further effect (lower panel, Fig. 6A). By contrast, NMDAR channel blockade by MK801increased apoptosis of scrambled HL60 cells. In the same HL60 cell line, shRNA knockdown of NMDAR was associated with lower expression of NADPH oxidase (isoform 2; NOX2; Fig. 6B) and the pro-survival kinase AKT (Fig. 6C), but not constitutively phosphorylated ERK (Fig. 6D). These data suggest that NMDAR-mediated signalling may contribute to neutrophil survival in a manner similar to that described in neurons.

### 3.7 Upregulation of GluN2B expression after systemic inflammation

**Fig. 7:**
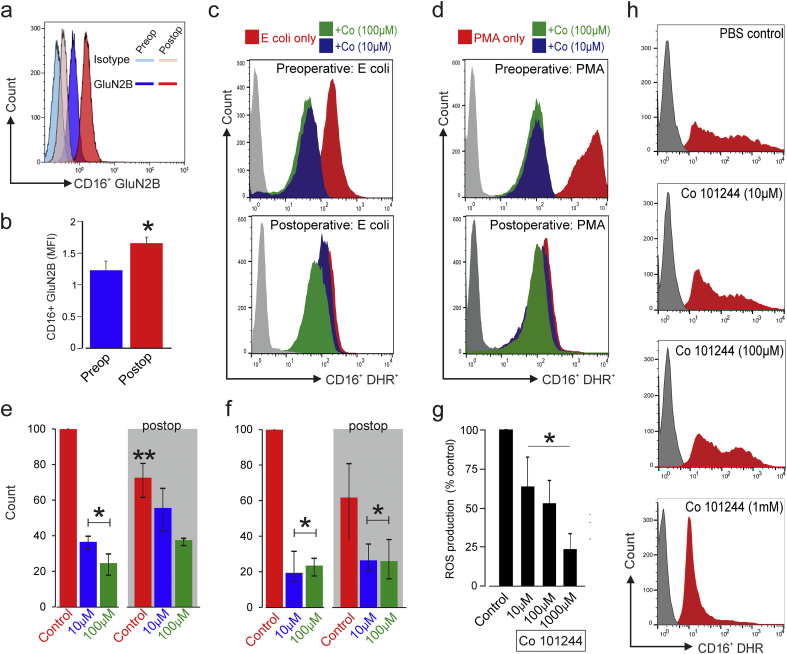
GluN2B subunit in systemic inflammation and neurologic-related bacterial infection. (A) Serial changes in GluN2B subunit expression in CD16+ primary human neutrophils obtained from surgical patients preoperatively and 48 h after surgery, quantified using flow cytometry. (B) Population data for CD16+ GluN2B expression in the perioperative period. (mean ± sem; p < 0.01, *t*-test; n = 6 patients assessed serially, preoperative versus postoperative). (C) Neutrophil ROS generation after E coli is added to whole blood from the same surgical patient preoperatively and 2 days after surgery, quantified by 123-dihydrorhodamine. PBS control, Co101244 at 10 μM and 100 μM was added to whole blood sample 10 min before *E. coli* stimulation. (D) Neutrophil ROS generation following addition of PMA to whole blood, from the same surgical patient preoperatively and 2 days after surgery, quantified by 123-dihydrorhodamine. PBS control, Co101244 at 10 μM and 100 μM was added to whole blood sample 10 min before *E. coli* stimulation. (E) Summary data (median, interquartile range) for preoperative and postoperative neutrophil responses to E coli in whole blood from 4 surgical patients following pre-incubation with PBS control, Co 101244 at 10 μM and 100 μM for 10 min prior to stimulation. * denotes p = 0.001 for drug versus saline control comparison; p = 0.035, ** denotes comparison between pre versus postoperative values; by repeated measures ANOVA (time × drug treatment). (F) Summary data (median, interquartile range) for preoperative and postoperative neutrophil responses to PMA in whole blood from 4 surgical patients following pre-incubation with PBS control, Co 101244 at 10 μM and 100 μM for 10 min prior to stimulation. * denotes p = 0.02 for drug versus saline control comparison; p = 0.07, comparison between pre versus postoperative values; by repeated measures ANOVA (time × drug treatment). (G) 10^6^ purified neutrophils (purity 98 ± 1%) from 4 healthy volunteers were incubated with GluN2B antagonist Co 101244 (0–100 μM shown) and *Pseudomonas fluorescens* (10^6^ cfu·ml^−1^). Asterisk denotes increasing doses of Co 101244 reduce ROS (quantified by 123-dihydrorhodamine fluorescence) in primary human neutrophils following co-culture with 10^6^ Pseudomonas fluorescens (mean ± sem, p = 0.011; ANOVA, F (3, 12) = 5.8). (H) Example data from one volunteer showing increasing doses of Co 101244 reduce ROS fluorescence in primary human neutrophils following co-culture with 10^6^*Pseudomonas fluorescens.*

In pro-inflammatory states, NMDAR expression is upregulated in non-neuronal, non-immune tissue such as cerebrovascular endothelium [46]. Moreover, in trauma patients, NMDAR gene expression is upregulated in neutrophils [18]. Therefore, we first examined whether GluN2B NMDAR protein expression changed in neutrophils following major surgery, where each sample was obtained from the same individual before and 48 h after major surgery (Fig. 7A). A significant increase in CD16+ neutrophil GluN2B expression was observed postoperatively, a period characterized by elevated oxidative stress (Fig. 7B). GluN2B upregulation was accompanied by reduced ROS generation in neutrophils stimulated by opsonised *E. coli* (Fig. 7C) and PMA (Fig. 7D) postoperatively (summarised in Fig. 5E–F).

### 3.8 GluN2B-mediated modulation of neurologic bacterial infection

*P. aeruginosa* accounts for up to ~18% of nosocomial meningitis cases after neurosurgery (O'Neill et al., 2006). We therefore further tested the clinical relevance of our findings by examining whether a closely related glutamate-producing bacillus, *Pseudomonas fluorescens*, modulated neutrophil inflammation in the presence of pharmacologic NMDAR GluN2B subunit blockade. Co 101244 dose-dependently reduced ROS production in primary human neutrophils obtained from 4 healthy volunteers (Fig. 7G) following 18 h incubation with *Pseudomonas fluorescens* (Fig. 7H).

## 4 Discussion

**Fig. 8:**
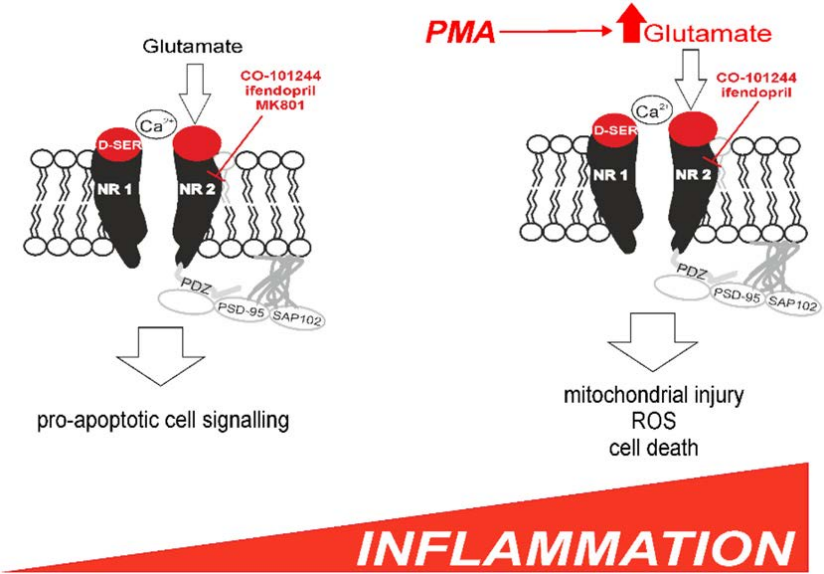
Summary of potential role for NMDAR/GluN2B signalling in modifying degree of ROS generation in activated neutrophils. Release of glutamate and co-agonist D-serine after activation by DAMPs/PAMPs promotes calcium influx to augment neutrophil ROS generation. Tonic levels of extracellular glutamate may play a protective role, since GluN2B antagonism promotes apoptosis in non-activated cells.

Glutamate has been implicated in generating tissue inflammation in several pathological models [47]. Glutamate is the metabolic precursor of the chief inhibitory neurotransmitter GABA that, via the GABA-A receptor, plays a well-established role in modulating macrophage function [48,49]. The principal finding of our studies is that glutamate may also contribute to several aspects of neutrophil function via activation of complexes formed by the NMDAR and associated scaffolding proteins. Neutrophil NMDAR-mediated signalling recapitulates many aspects of neuronal NMDAR biology (summarised in Fig. 8). Our translational clinical data using in vivo murine and ex-vivo human models suggest a role for NMDAR gating in modulating the neutrophil inflammatory response to infections relevant to the CNS.

A role for glutamate and (indirectly) NMDAR-mediated signalling has been implicated in a broad range of immunology-related areas, including macrophage biology [11,50,51], lymphocyte function [52], and neutrophil-mediated lung injury [53]. However, a functional role for NMDAR in neutrophils has chiefly been inferred through pharmacologic antagonist studies [54]. Off-target effects of various antagonists including K^+^ channel inhibition [55] and non-specific (anaesthesia-like) effects on other membrane targets [56] may account for many findings attributed to (potential) NMDAR involvement [51,54,[57], [58], [59]]. However, the general anesthetic isoflurane, which suppresses neuronal NMDAR activity [60], attenuates zymosan-induced lung injury by reducing neutrophil NMDAR/TLR2 mediated NADPH oxidase generation of superoxide [10]. A further precedent for the involvement of glutamate in innate immune regulation has been proposed through the observation that glutamate released by activated human polymorphonuclear cells decreases human brain endothelial barrier protection, mediated by endothelial mGluRs [12]. Akin to previous observations on the location of glutaminase, [61]. we found that serine racemase is present on the neutrophil cell surface and release of d-serine increases in response to PMA challenge. These data suggest that d-serine acts as a co-agonist recruited to maintain NMDAR activity in neutrophils, rather than glycine which inhibits the neutrophil respiratory burst [62]. Thus, the necessary machinery is in place in human neutrophils for glutamate generated via glutaminase to activate NMDAR in an auto- and paracrine manner.

As first demonstrated in immune cells [9], the NADPH oxidase-2 (NOX2) isoform is the major source of neuronal ROS produced following NMDAR activation by glutamate. In neurons, calcium influx triggers release of superoxide by activating neuronal NADPH oxidase [7]. Disruption of the GluN2B NMDAR subunit binding to the scaffolding protein postsynaptic density protein 95 (PSD-95) blocks phosphorylation of the critical p47^phox^ subunit of NADPH oxidase [29]. Depending on the source, and concentration of extracellular glutamate, GluN2B subunit-containing NMDA receptors contributes to neuronal survival [45]. Therefore, to further dissect the role of NMDAR-mediated signalling in neutrophil biology, we used GluN2B NMDAR subunit specific small molecule inhibitors, as well as genetic models of NMDAR subunit and scaffolding protein deficiency that were originally developed to understand the role of NMDARs in the brain. The results obtained in this study support the hypothesis that the neural and immune systems share key signalling mechanisms of functional relevance [63].

Further insights were afforded from the maturation of HL60 cells into a neutrophil-like phenotype, which was characterized by increased ROS generation (in response to PMA challenge) and a parallel increase in GluN2B expression. The role of the GluN2B subunit in generation of superoxide by the neuronal NADPH oxidase is well established [7]. The NOX2 isoform of NADPH oxidase is the predominate isoform expressed by both neurons and neutrophils [9]. Since GluN2B-specific antagonists similarly reduced ROS production in response to PMA and bacterial challenge in primary neutrophils, this appears to be a conserved feature of both neural and immune systems. Furthermore, we observed a direct pathophysiologic correlate of these findings, in that ROS generation was reduced in highly purified neutrophils co-incubated with a GluN2B antagonist and *Pseudomonas fluorescens*, a glutamate producing bacteria. Extracellular calcium augments neutrophil ROS generation following priming with pro-inflammatory cytokines [64]. Augmentation of the neutrophil respiratory burst by the presence of extracellular calcium recapitulates the calcium-dependent role described for NMDA-induced NOX2 activation in neurons with NMDA receptors [7,65]. In further support of this hypothesis, genetic ablation or small molecule inhibition of PSD-95 attenuated ROS production, paralleling the role of these pivotal scaffolding proteins in neurons. The combination of ubiquitous PKC activation through inflammatory ligands ungating the NMDAR, and excess release of glutamate, appears to facilitate this regulatory role for extracellular calcium.

The finding that systemic inflammation (after surgery) is associated with increased expression of NMDAR in neutrophils obtained from patients postoperatively is consistent with the upregulation of NMDAR subunit expression reported in microarray analyses of neutrophils in patients within 96 h of sustaining major trauma [18]. Specifically, microarray analysis showed that GluN1 expression was increased ~5-fold, which was accompanied by ~22-fold upregulation of NMDA receptor-regulated gene 1 (NARG1).This expression pattern is in contrast to the reduced expression of NMDA receptor-regulated genes during early postnatal development, when GluN1 receptor expression is up-regulated [66]. As reported in trauma patients, the GluN2B upregulation was associated with neutrophil dysfunction [67]. The preoperative versus postoperative comparison within the same patient adds further strength to these findings, and is consistent with previous reports that inhalational anaesthesia interacts with the NMDAR in neutrophils [10]. The upregulation of the GluN2B receptor observed in neutrophils obtained from the systemic inflammatory milieu of the postoperative environment mirror similar findings in neurons, where tumour necrosis factor (TNF)-alpha-induces a rapid increase in the surface density of GluN1B subunit in hippocampal neurons [68].

In summary, our results indicate that the auto/paracrine release of glutamate and d-serine acting on NMDARs contributes to several regulatory mechanisms involved in neutrophil biology in a manner analogous to that described in neurons. These data provide further potential insights into why recruitment of neutrophils may further exacerbate brain injury through the release of glutamate triggering pathophysiologic NMDAR signalling. Shared mechanisms exhibited by circulating cells that are readily available from patients with, or at risk from, neurological disease suggest a potential role for this paradigm to further understanding of neuropathology.

## Author contributions

GLA, AVG AGDA designed the experiments and wrote the manuscript; AGDA, AH, ET, DC, JS, AVG, GLA, VK,TN performed the experiments; SL, DC, GLA analyzed clinical data; SNG provided mutant murine tissue; GLA supervised the project.

## Declaration of Competing Interests

Funding bodies played no role in the design of the study and collection, analysis, and interpretation of data or in writing the manuscript. GLA has patent pending in conjunction with UCL Business (WO/2016/005753/PCT/GB2015/051986) based on detection of molecules released during inflammation.

## Funding sources

GLA: supported by an Academy of Medical Sciences/Health Foundation Clinician Scientist award (CSF3-Ackland), British Journal of Anaesthesia and Royal College of Anaesthetists basic science fellowship, British Oxygen Company grant. AVG: supported by a Wellcome Trust Senior Fellowship. SGNG: supported by Wellcome Trust and Medical Research Council. AH: supported by Grand Challenges UCL award. JS: supported by a British Journal of Anaesthesia/Royal College of Anaesthetists basic science PhD studentship. SL: supported by UK NIHR Clinical Academic Fellow award. AGDA: no conflicts of interest. ET: no conflicts of interest. DC: no conflicts of interest.

## Appendix A Supplementary data

Supplementary material Image 1
