# Supplementary material for: NMDA receptor modulation of glutamate release in activated neutrophils
Source: eBioMedicine. 2019 Aug 8;47:457–69. doi: 10.1016/j.ebiom.2019.08.004 (PMC6796524; doi:10.1016/j.ebiom.2019.08.004)
Supplement: Supplementary file 1 — Supplementary material [file mmc1.pdf]

SUPPLEMENTAL DIGITAL CONTENT

NMDA receptor modulation of glutamate release in activated neutrophils.

Contents

Supplementary Figure 1. Flow cytometry and immunoblotting for NMDAR-related proteins.3

Supplementary Figure 2. NMDAR and AMPA blockers 4

Supplementary Figure 3. Bone marrow derived murine neutrophils.. 5

Supplementary Figure 4. Neutrophil apoptosis, but not activation, after extracellular addition of NMDA and D-serine at concentrations detected after neutrophil activation with bacteria or PMA. 6

Supplementary Figure 5. ERK phosphorylation in presence/absence of NMDAR GluN2B inhibitor 7

Supplementary Figure 6. NMDAR GluN2B antagonist does not alter phosphorylation of PKC. 8

Supplementary Figure 7. Anti-bacterial actions of NMDAR GluN2B antagonist 9

Materials and Methods 10

  Reagents 10

  Purification of cells 10

  Cell lines 10

  Mice 11

  Peritoneal neutrophil harvest 11

  Patient study 11

Flow cytometry 12

Neutrophil oxidative burst.. 13

Neutrophil oxygen consumption 13

Neutrophil phagocytosis.. 14

Bacterial culture experiment. 14

Microelectrode biosensors... 15

Immunoblots.. 15

Short hairpin gene knockdown... 16

SiRNA gene knockdown in primary human neutrophils. 16

Real time PCR.. 17

Additional references 18

Supplementary Figure 1. Flow cytometry and immunoblotting for NMDAR-related proteins.

(**A**) Whole blood human primary human neutrophils, intracellular staining. (**B,C**) Representative flow cytometry plot showing isotype control quadrants, fluorescence minus one gating for APC/FITC intracellular staining for CD16+ GluN2B expression in purified isolated human primary human neutrophils. (**D)** Representative flow cytometry plot showing isotype control quadrants and CD16+ GluN1 expression in purified isolated human primary human neutrophils. (**E**) Immunoblots for GluN1 (NMDAƐ1 (H-54); sc-9056), SAP102 (sc-134400) and PSD95 (H-40; sc-28941) in mouse brain cortex and isolated purified primary human neutrophils. β-actin served as loading control. (**F**) Immunoblots for GluN1 (NMDAƐ1 (H-54); sc-9056), GluN2B, SAP102 (sc-134400) and PSD-95 (H-40; sc-28941) in mouse brain cortex, undifferentiated and differentiated HL60 cells (1.25%DMSO, for 6 days). β-actin served as loading control. (**G**) RT-PCR showing SAP102 mRNA in primary human neutrophils, undifferentiated (ud) and HL60 cells differentiated with DMSO for 3-6 days, referenced to human neuroblastoma LN229 cell line (which expresses NMDAR in low quantities; mean±sem). N=3-5 group; 3 independent experiments.

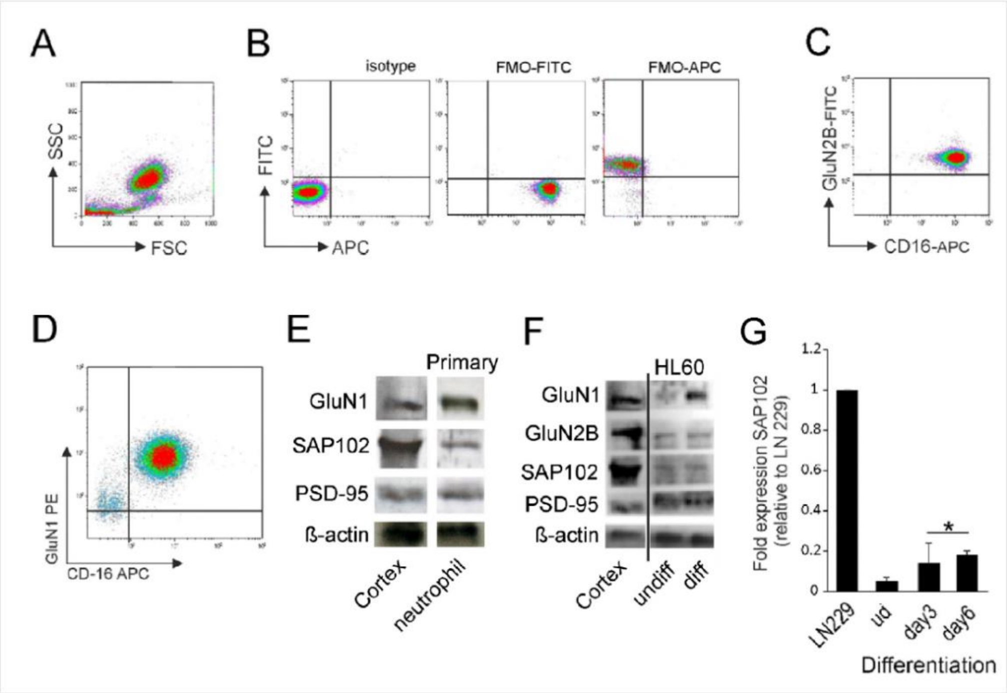

Supplementary Figure 2. NMDAR and AMPA blockers

Suppl. Figure 2. (**A**) Forward and side scatter characteristics of magnetic bead positively selected primary human neutrophils. (**B**) Quantification of apoptosis following bead isolation. (**C**) Typical purity of primary human isolated neutrophils following bead isolation as defined by CD16+ cells. (**D**) Co 101244 and MK801 reduce ROS release from CD16+ neutrophils following PMA-stimulation. (**E**) Co 101244 and ifendopril reduces ROS release from CD16+ neutrophils following *E coli* and PMA-stimulation. **(F)** Summary data for CO 101244 and ifendopril effect on ROS release from CD16+ neutrophils following *E coli* and PMA-stimulation. (mean±sem, n=3-7/group; *p<0.01, ANOVA). (**G-J**) Quantification of ROS generation (DHR-FITC) in highly purified peritoneal Ly6G+ neutrophils (**J**) obtained 3h after intraperitoneal injection of zymosan in C57B/6 mice, using unstained (quadrant), isotype controls (**G**) and fluorescence minus one gating (**H-I**). **(K)** Blockade of AMPA (α-amino-3-hydroxy-5-methyl-4-isoxazolepropionic acid) receptors using CNQX (100μM) has no effect on PMA-induced ROS production. (**L**) Using the dihydroethidium ROS probe, fluorescence is reduced by Co 101244 in CD16+primary human neutrophils. All summary data mean±sem.

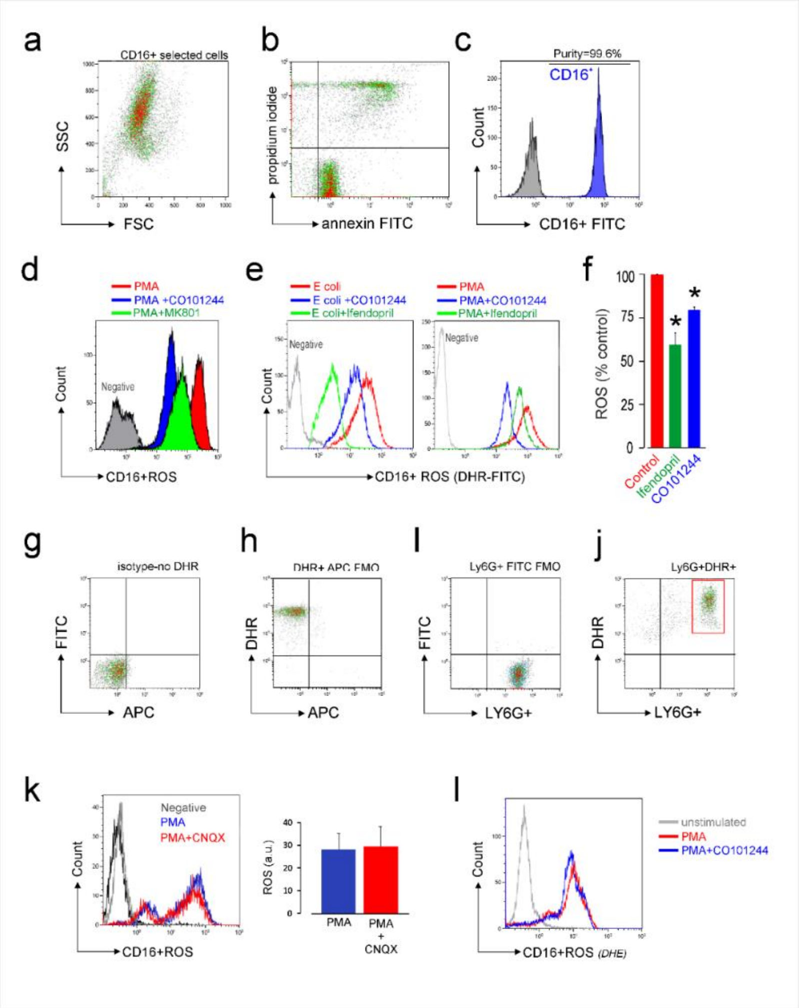

Supplementary Figure 3. Bone marrow derived murine neutrophils.

(A) Unstained bone marrow-derived neutrophils. (**B**) Viable bone marrow-derived neutrophils gated by 7AAD fluorescence. (**C**) Isotype control for bone marrow-derived neutrophils, quadrants set for unstained cells. (**D**) Fluorescence-minus-one gating for APC+ cells. (**E**) Fluorescence-minus-one gating for PE+ cells. (**F**) Confirmation of $Ly6G^{+}CD11b^{+}Ly6C^{-}$ neutrophils.

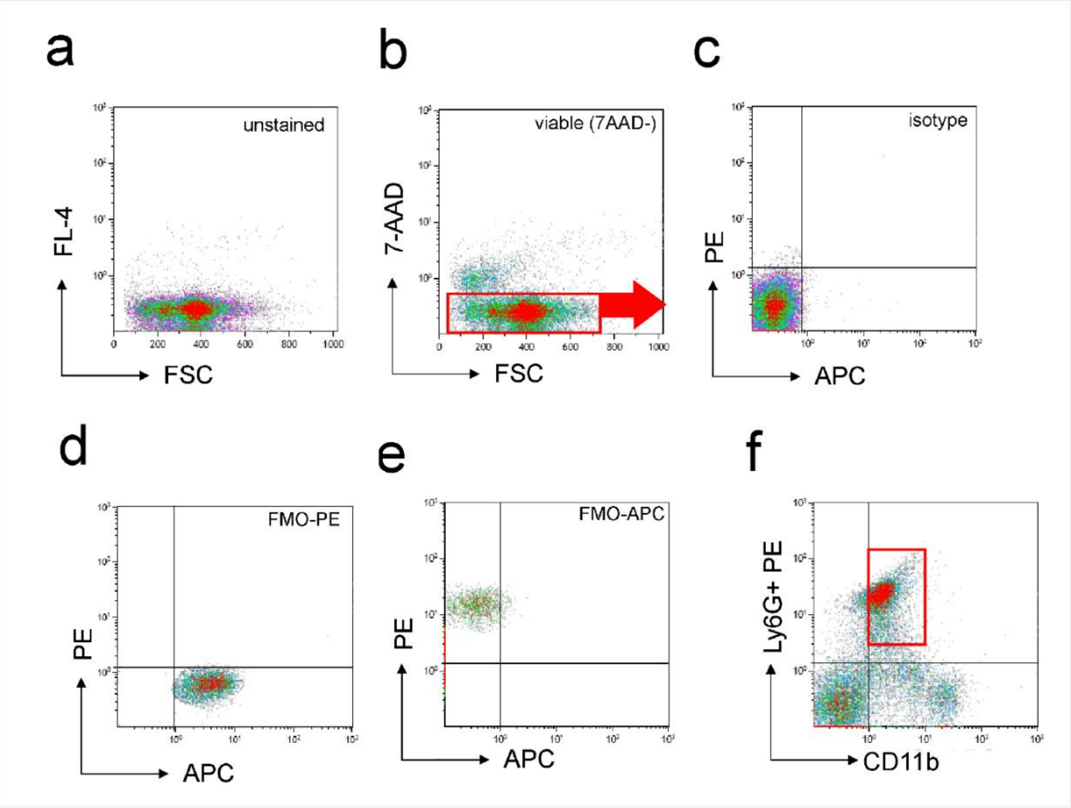

Supplementary Figure 4. Neutrophil apoptosis, but not activation, after extracellular addition of NMDA and D-serine at concentrations detected after neutrophil activation with bacteria or PMA.

NMDA (20 or 50µM) applied to primary human neutrophils for 10 minutes, in combination with its co-agonist D-serine (5µM), induces rapid ~20% apoptosis (loss of CD16), but not appreciable activation (increase in CD11b). B. Summary graph from three individual experiments showing proportion of primary neutrophils obtained from 3 separate subjects that lose CD16 expression (asterisks denote $p<0.01$, comparing NMDA/D-serine versus control neutrophils; paired t-test) and activation (increase in CD11b median fluorescence intensity). C. Histogram showing ROS in untreated primary human neutrophils and after addition of NMDA (20µM) and its co-agonist D-serine (5µM). D. Summary data from four individual experiments with primary human neutrophils ($p<0.01$, by ANOVA). The ROS inhibitor diphenyleneiodonium chloride (10µM) lowered ROS in the presence or absence of NMDA/D-serine (asterisks denote $p<0.01$, comparing DPI versus neutrophils no treated with DPI, compared by paired t-test).

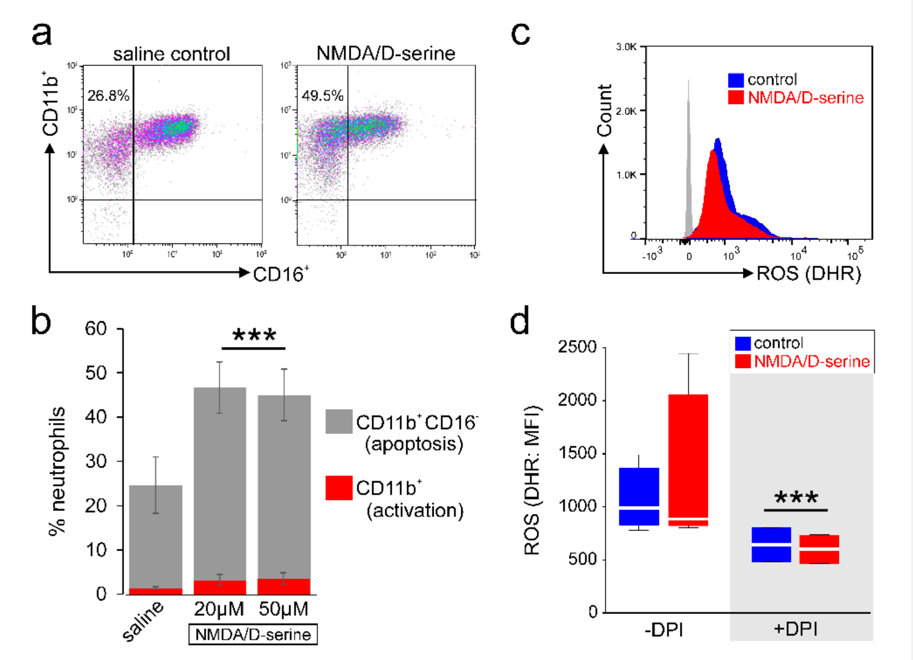

Supplementary Figure 5. ERK phosphorylation in presence/absence of NMDAR GluN2B inhibitor

GluN2B antagonism (Co 101244, 100µM) reduces PMA-induced phosphorylation of ERK in primary human neutrophils, as assessed by immunoblot in 5 separate individuals. Graph shows median (25-75th centile) fold change from baseline phosphoERK/total ERK ratio (p=0.06, by repeated measures ANOVA).

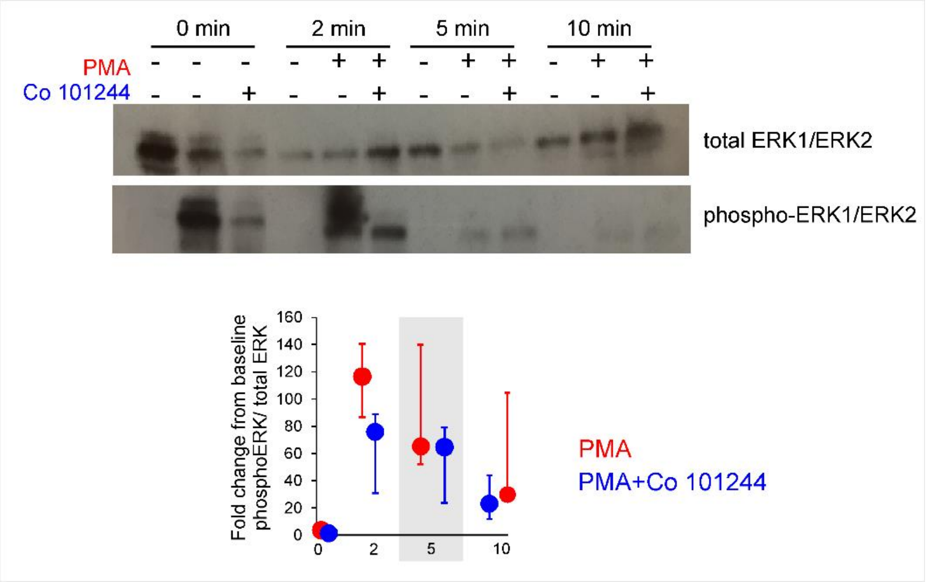

Supplementary Figure 6. NMDAR GluN2B antagonist does not alter phosphorylation of PKC.

(a) Representative immunoblots of similar PKC phosphorylation (phospho-MARCKS (Ser152/156) antibody), 2 minutes after PMA activation of primary human neutrophils obtained from 2 healthy volunteers, in presence/absence of NMDAR GluN2B antagonist CO-101244. (b) Knockdown of GluN2B does not affect PKC phosphorylation in primary human neutrophils. (c) Similar PKC phosphorylation 2 minutes after PMA activation of primary human neutrophils obtained from 2 healthy volunteers, in presence/absence of NMDAR GluN2B antagonist CO-101244. (d) Summary data for 3 individual experiments as detailed in panel c, using primary neutrophils from three subjects (mean (SD)).

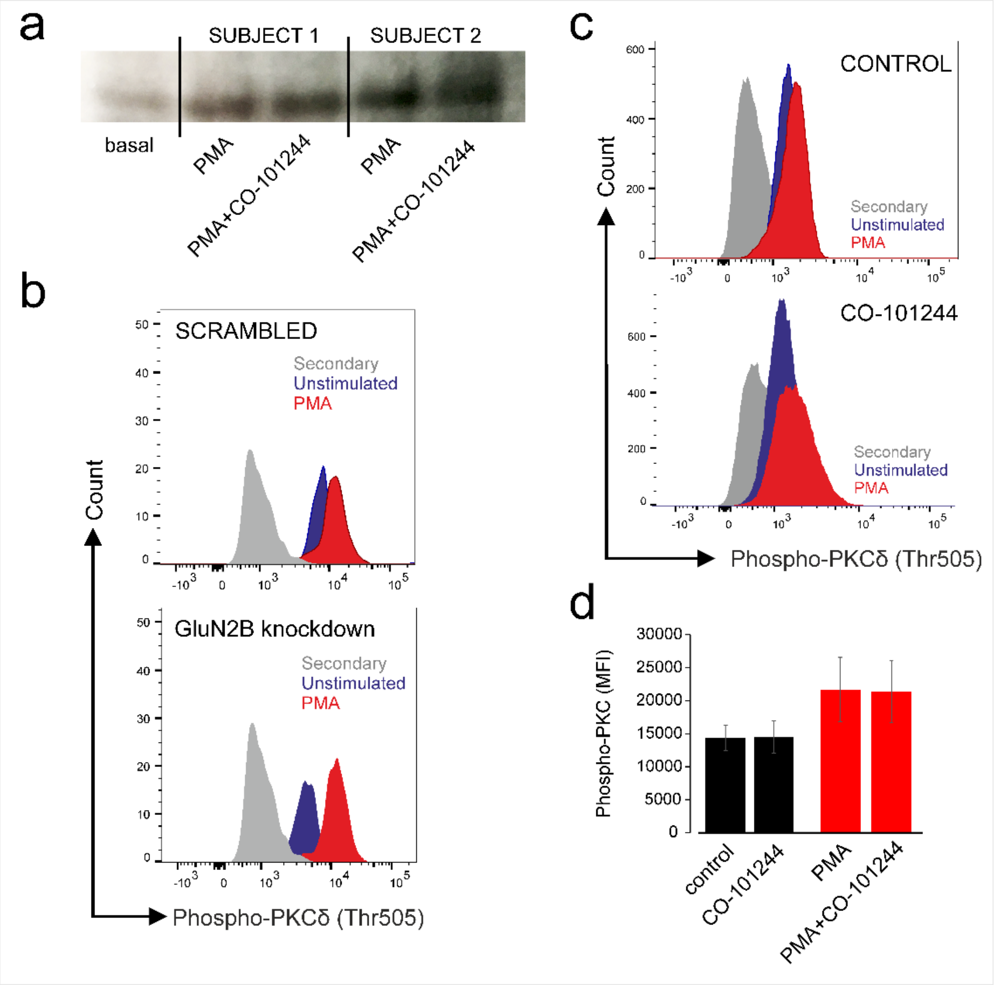

Supplementary Figure 7. Anti-bacterial actions of NMDAR GluN2B antagonist

(**a**) Purity of primary human neutrophils (purity >98%) obtained from healthy volunteers, n=4). Unstained/isotype control shown in black; CD16+ cells –red. (**b**) ROS generation by human neutrophils following incubation with *P. fluorescens* in the absence and presence of GluN2B blockade with Co 101244. Grey shaded histogram denotes DHR- (unstained) cells. Concentration of Co 101244 shown; CD16+ DHR+ median fluorescence intensity values shown above each line.

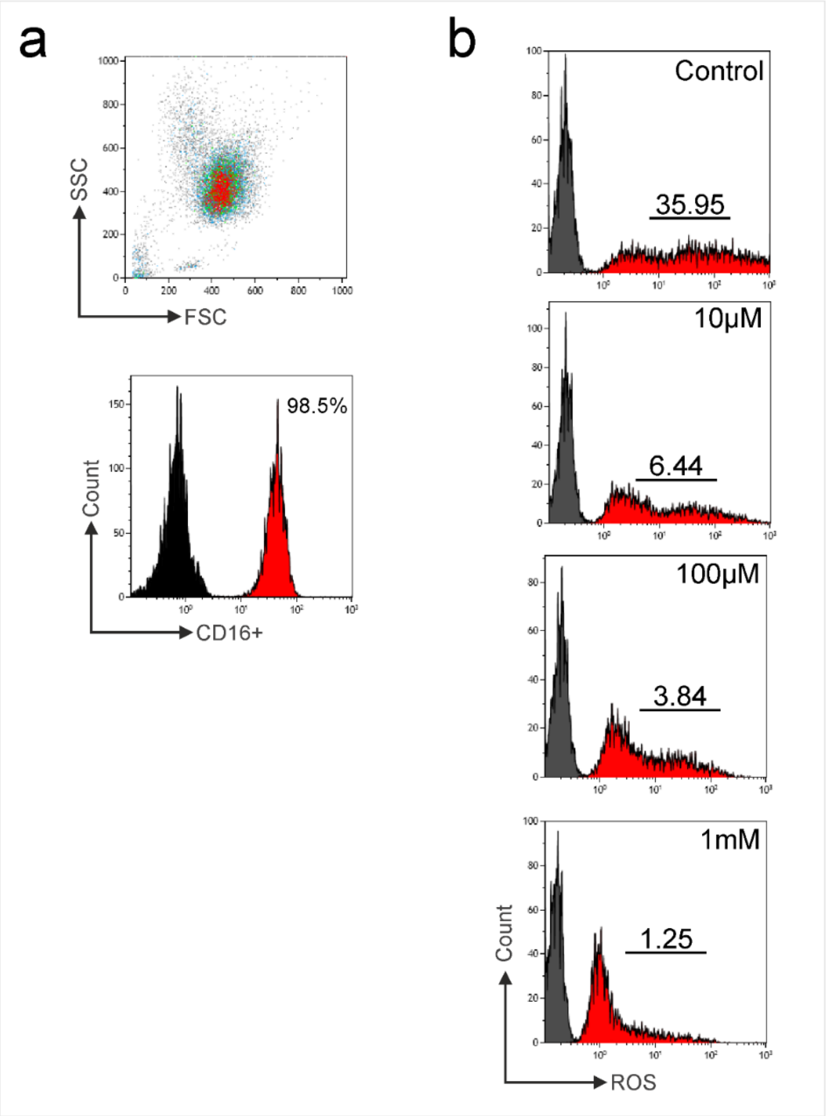

Materials and Methods

Reagents

Reagents were acquired from Sigma-Aldrich (Poole, UK) unless stated otherwise. Co 101244 hydrochloride (10^{-9} to10^{-3}M; vehicle water), ifenprodil hemitartrate (50µM; vehicle water), ZL006 (10-50µM; vehicle DMSO, final concentration <0.1%), 6-cyano-7-nitroquinoxaline-2,3-dione (CNQX; 100 µM; Tocris Bioscience, Bristol, UK).

Purification of cells

Granulocytes were purified from human blood obtained from healthy volunteers of either gender, aged 25-45y and free of acute or chronic inflammatory disease (MREC: 11/H0722/3). Using Ficoll density centrifugation (Ficoll paque plus, GE Healthcare, Amersham UK), >97% purity was achieved as quantified by CD16+ CD14- surface staining. Highly purified neutrophils were obtained using Ficoll separated blood samples followed by positive selection with CD16 microbeads (Miltenyi Biotec, Bisley, UK) achieving >98.5% purity as assessed by CD16+ surface staining.

Cell lines.

HL60 cells (ATCC number: CCL-240™) were differentiated into neutrophils according to published methodology.(1) The LN229 glioma cell line, of which ~3% strongly express NMDA-R on the cell surface (2), was a kind gift from the laboratory of Professor John Garthwaite, University College London.

Mice

All experiments were performed in accord with the UK Animals (Scientific Procedures) Act (1986) and ARRIVE guidelines. Male and female mice were used, 7-12 weeks old. Each mouse served as their own control in experiments. PSD-95$^{-/-}$ were provided by SG.(3) Generation of PSD-95$^{-/-}$ mice have been described previously.(3) C57B/6 mice were obtained from Charles River UK. Bone marrow was extracted from age and gender-matched mice. Non-viable cells were excluded by 7AAD staining, and neutrophils were identified by Ly6G^{+}Ly6C^{-}CD11b^{hi} staining.

Peritoneal neutrophil harvest

Zymosan (1mg.g^{-1}; Sigma, UK) was injected intraperitoneally in C57B/6 wild-type mice of either gender (age 6-8 weeks), and treated with either sterile saline or Co 101244 (100μM ex vivo). Peritoneal lavage with 5ml ice cold PBS was undertaken 3h after zymosan injection. Peritoneal exudate cells were pelleted by centrifugation (300 × *g*, 5 min). Non-viable cells were excluded by 7AAD staining, and neutrophils were identified by Ly6G^{+}Ly6C^{-}CD11b^{hi} staining. ROS activity was identified in viable, co-stained Ly6G+ cells.

Patient study

Written consent was obtained from male and female surgical patients (age>45y) to use whole blood samples for neutrophil experiments quantifying GluN2B expression and ROS generation in whole blood (MREC: 11/H0722/3).

Flow cytometry

Neutrophils were identified using forward and side scatter characteristics, in combination with specific cell surface antigen markers. After blocking, cells were stained using antibodies (all Miltenyi Biotec, Bergisch Gladbach, Germany) against human CD16 (clone VEP13;), CD14 (clone TUK4) and CD11b (clone M1/70.15.11.5) or the appropriate isotype control. Serine racemase (clone 7E8) antibody was acquired from Abcam (Cambridge, UK). NMDAR subunits and associated scaffolding proteins were quantified with appropriate secondary antibodies or isotypes as controls, using validated antibodies as indicated: NMDAε1 antibody (clone H-54 rabbit polyclonal IgG; sc-9056, Santa Cruz)(4); NMDAζ1 Antibody (clone C-20; goat polyclonal IgG, sc-1467 (conjugated with phycoerythrin), Santa Cruz)(5); GluN2B-FITC (IgG linear peptide corresponding to mouse NR2B; FCABS332F, Merck Millipore); PSD-95 antibody (clone H-40, rabbit polyclonal IgG, sc-28941; Santa Cruz)(6); SAP102 (NE-dlg, 7H11; sc-134400, Santa Cruz). PKC phosphorylation was quantified using Phospho-PKCδ (Thr505) Antibody (Cell signalling Technology, #9374). For mouse experiments, the following antibodies (all Miltenyi Biotec) were used: GR-1 (clone RB6-8C5), CD16-32 (clone 93), CD11b (clone M1/70.15.11.5). Intracellular staining was performed using BD Cytofix/Cytoperm™ Fixation/Permeabilization Solution Kit, (BD Biosciences, Oxford, UK) with either isotype or secondary antibodies serving as controls. Intracellular staining was performed using the BD Biosciences Fix/perm kit/protocol. To set the gates, flow cytometry dot plots were based on comparison with isotype controls, fluorescence minus one (FMO), permeabilized and unpermeabilized unstained cells. Data were acquired with a Cyan ADP fluorescent-activated cell sorter (Beckman Coulter, MI USA) or FACSCalibur machine, captured using Summit (Beckman Coulter, MI USA) or CellQuest software and analyzed with Kaluza software (Beckman Coulter, MI USA).

Neutrophil oxidative burst

Respiratory burst activity was assessed using the Phagoburst® assay(7) in isolated neutrophils in phosphate buffered saline, citrated or heparinized peripheral venous blood samples (100µl), with a minimum n=4 subjects, performed in at least 3 separate experiments. Opsonized *Escherichia coli (3x10^6 bacteria)***,** FMLP(1-5µM) or Phagoburst® washing solution (negative control) were added to blood or isolated neutrophils for 10 min at 37°C in a water bath. To elicit a maximal response, PMA (0.9µM), Dihydrorhodamine 123 (DHR; 20 µl) was then added to each sample and incubated for another 10 min at 37°C in a water bath. In the presence of reactive oxygen species (ROS), DHR is converted into a green fluorescent dye. For lysis, the samples were incubated with lysis solution for 20 min at room temperature. Cells were washed with Phagoburst® washing solution. Oxidative burst activity was quantified by median fluorescence intensity and number of FITC-positive cells within the gated neutrophil population. Dihydroethidium (5µM) was also used as a further ROS probe for whole blood experiments.

Neutrophil oxygen consumption

Oxygen consumption was measured using the Seahorse XF instrument (Seahorse Biosciences, North Billerica, MA). Cells were seeded into wells of 24 well-tissue culture plates coated with CellTak™ cell adhesive, at a density of 1.5x10^6 cells per well in unbuffered XF assay media at pH 7.4, supplemented with 25 mM glucose (Sigma-Aldrich, St. Louis, MO) and 1 mM sodium-pyruvate and 1 mM glutamax (Invitrogen, Carlsbad, CA). Cells were incubated for 1 h at 37°C ambient O_2 and CO_2 concentration before measurements were taken. Respiration was measured in 4 blocks of 3 times for 3 minutes. The first block measured the basal respiration rate. Drugs (Co 101244; 25-250µM) or control (unbuffered Seahorse medium) injections were then injected, 10 minutes prior to injection of vehicle

control (DMSO) or PMA (500ηM; Sigma-Aldrich) which was added to stimulate NADPH oxidase activity. Final measurements were performed over a period of 60 minutes. Experiments undertaken with a minimum n=3, performed in 3 separate experiments.

Neutrophil phagocytosis

200µl heparinized whole peripheral blood was incubated with 40µl opsonized FITC-labeled *Escherichia coli* for 10 min at 37°C in a water bath. Samples remaining on ice served as negative controls. To stop phagocytosis, 200 µl Phagotest® quenching solution was added to each sample at the end of the incubation time. Samples were then washed with 3 ml Phagotest® washing solution twice and lysed with 2 ml Phagotest® lysis solution for 20 min at room temperature. PI excluded dead/non-viable cells. After a further wash, neutrophils were either processed immediately or stained with anti-CD16–APC for 20 min at 4°C, before the final assay step. Neutrophil subsets were defined according to their scatter properties and CD16+ expression. Experiments with a minimum n=4, performed in 3 separate experiments.

Bacterial culture experiment.

Pseudomonas fluorescens (*P. fluorescens*), a glutamate-producing bacillus, preferentially proliferates in mouse organ homogenates at 4˚C.(8) We cultured and incubated *P. fluorescens* (10^6 cfus/ml) with $3x10^6$ highly purified (97±1%) primary neutrophils ($10^6.ml^{-1}$) obtained from healthy volunteers (n=4) for 18h in Dulbecco's Modified Eagle's medium (without antibiotics). Co 101244 (10-1000uM) or vehicle control were added to these samples. This strain of *P. fluorescens* was resistant to penicillin, cephalosporins and clindamycin (Royal Veterinary College Diagnostic Laboratories, North Mymms, Herts UK).

Microelectrode biosensors

The design and operation of enzyme-based glutamate biosensors have been described in detail previously(9, 10). The biosensor contained glutamate oxidase entrapped within a matrix around a Pt wire (diameter 50µm, length 1mm). Glutamate oxidase converts glutamate to α-ketoglutarate with production of NH_3 and H_2O_2. The glutamate sensor relies on the amperometric detection of H_2O_2 produced within the thin enzymatic layer around the microelectrode tip, achieving a 10–90% response time of <10s. Electrochemical sensors respond not only to the analyte of interest, but also to any other electroactive species in the milieu. In each experiment, we controlled for the release of non-specific electroactive interferents by using a dual recording configuration: in addition to the glutamate sensor, a null sensor lacking enzymes but otherwise identical in size and shape was placed into a well containing $1.5x10^6$ neutrophils ml^{-1} in PBS, and maintained at 37°C through a heating pad. Sensors were calibrated immediately before and after the recordings to test whether they retained sensitivity. During the recordings, sensors lost <10% of their initial sensitivity. To convert changes in sensor current to changes in analyte concentrations, the mean of the initial and final calibrations was used.

Immunoblots.

Neutrophil or HL60 pellets were washed with PBS and resuspended in modified RIPA lysis buffer (50mM Tris-HCL pH 7.4, NP-40 1% v:v , 0.25% Na-deoxycholate, 150mM NaCl, 1mM EDTA) with PMSF 1mM and cocktail of Protein Inhibitors (aprotinin, leupeptin, pepstatin,1µg/ml each). Lysates were sonicated at ten pulses of low intensity and centrifugated at full speed 10 minutes at 4°C and supernatants recovered. Loading buffer was added to the samples (2% SDS, 62.5mM Tris pH 6.8, 10% glycerol, 0.01% bromophenol

blue, 1.25% β-mercaptoethanol) and boiled 5 min before loading in the 10% acrylamide gel. Transfer to PVDF membrane (Hybond-Amersham, UK) was undertaken in a BioRad semi dry apparatus using a classical Tris-Glycine buffer plus 0.1% SDS. Membrane was blocked with 5% dry milk in PBS-0.1% Tween-20. The membranes were incubated with the following primary antibodies (from Santa Cruz Biotechnology, Heidelberg, Germany, unless otherwise stated): GluN1 (sc-9056), NE-dlg (SAP102; sc-134400), PSD-95 (sc-28941), ERK (1:1000; Cell Signaling #4695), phospho-ERK (1:1000; Cell Signaling #4370),
AKT (1:1000; Cell Signaling #9272), phospho-AKT (S473) (1:1000; Cell Signaling #9271), NOX-2 (1:1000; Abcam, ab129068), phospho-MARCKS (Ser152/156) Cell Signaling #2741). Loading control used was GAPDH (1:1000; Cell signaling). Secondary antibodies (1:2000; Dako, Stockport, UK or Cell Signaling) were rabbit anti mouse HRP or goat anti-rabbit HRP, as indicated. Membranes were developed using ECL™ reagent and Hyperfilm ECL (Amersham, UK).

Short hairpin gene knockdown.

GluN1B was knocked down in HL60 cells prior to differentiation into neutrophil-like cells, using five short hairpin RNA lentiviral clones expressing the pLKO.1-puromycin vector (MISSION shRNA, Sigma, Poole UK). Stable gene silencing is achieved in this system by using the puromycin selectable marker. We compared the phenotype of the clone that resulted in the highest knockdown of GluN1B with scrambled constructs, in addition to untransfected cells.

SiRNA gene knockdown in primary human neutrophils.

SiRNA knockdown targeted against GluN1 and GluN2B in 2-5x10^6 primary human neutrophils (11)was undertaken using electroporation (Amaxa nucleofector II, Lonza).

Negative control siRNA, siCONTROL Non-Targeting siRNA (Control-siRNA), contains at least four mismatches to any human, mouse, or rat gene. siRNAs sequences were:

GRIN1 siRNA human Alex647: 5Alex647N/CAAGGAGGAGUUCACAGUCAA

GRIN2 siRNAhumanAlex647: 5Alex647N/CACGGCCAAGAACAUGGCUAA

si hmr Scramble Alexa: 5Alex647N/UAAGGCUAUGAAGAGAUAC

Real time PCR.

RNA from neutrophils pellets was extracted using the RNAeasy kit according to the manufacturers protocol (Qiagen, Crawley, UK). RNA integrity and concentration was tested on a ND-1000 spectrophotometer (Nanodrop, Wilmington, USA). Real-time PCR was executed in two steps. Reverse transcriptase was performed using the standard protocol of the Taqman kit (Roche, Burgess Hill, UK) from 2ug of RNA using random hexamers in a final volume of reaction of 50ul. For the 2nd step 0.5ul of cDNA reaction product was used per well and in triplicate. The amplification product was monitored by incorporation of SYBR green (Roche, Burgess Hill, UK). Real time PCR was carried out using Eppendorf Realplex apparatus (Eppendorff, Stevenage, UK). The following primer sequences (Sigma-Aldrich, Poole, UK) were used, as reported previously for GluN2B(12): NR2B forward: AGCTTCACGCATTCTGACTG, NR2B reverse: CTTGGTACACGTTGCTGTCC; GAPDH forward: TGCACCACCAACTGCTTAGC, GAPDH reverse: GGCATGGACTGTGGTCATGAG; SDH forward: CAAACAGGAACCCGAGGTTTT, SDH reverse: CAGCTTGGTAACACATGCTGTAT Expression values of NR2B were normalised to GAPDH (HL60 differentiation) or SDH (shRNA cells) and are reported in units of $2^{-\Delta CT}$ related to expression in the LN229 cell line, were ΔCt is the difference in Ct values between NR2B and SDH/GADPH in the same sample.

Additional references

1. Collins SJ, Ruscetti FW, Gallagher RE, et al. Normal functional characteristics of cultured human promyelocytic leukemia cells (HL-60) after induction of differentiation by dimethylsulfoxide. J Exp Med 1979;149(4):969-974.
2. Mittelbronn M, Harter P, Warth A, et al. EGR-1 is regulated by N-methyl-D-aspartate-receptor stimulation and associated with patient survival in human high grade astrocytomas. Brain Pathol 2009;19(2):195-204.
3. Cuthbert PC, Stanford LE, Coba MP, et al. Synapse-associated protein 102/dlgh3 couples the NMDA receptor to specific plasticity pathways and learning strategies. J Neurosci 2007;27(10):2673-2682.
4. Wang HY, Bakshi K, Frankfurt M, et al. Reducing amyloid-related Alzheimer's disease pathogenesis by a small molecule targeting filamin A. J Neurosci 2012;32(29):9773-9784.
5. Wang YB, Wang JJ, Wang SH, et al. Adaptor protein APPL1 couples synaptic NMDA receptor with neuronal prosurvival phosphatidylinositol 3-kinase/Akt pathway. J Neurosci 2012;32(35):11919-11929.
6. Wu LX, Sun CK, Zhang YM, et al. Involvement of the Snk-SPAR pathway in glutamate-induced excitotoxicity in cultured hippocampal neurons. Brain Res 2007;1168:38-45.
7. Prince HE, Lape-Nixon M. Influence of specimen age and anticoagulant on flow cytometric evaluation of granulocyte oxidative burst generation. J Immunol Methods 1995;188(1):129-138.
8. Tatara Y, Terakawa T, Yamagata Y, et al. Pseudomonas fluorescens proliferates in a mouse organ homogenate at low temperature. Int J Mol Med 2008;21(5):621-626.
9. Gourine AV, Dale N, Korsak A, et al. Release of ATP and glutamate in the nucleus tractus solitarii mediate pulmonary stretch receptor (Breuer-Hering) reflex pathway. JPhysiol 2008;586(16):3963-3978.
10. Tian F, Gourine AV, Huckstepp RT, et al. A microelectrode biosensor for real time monitoring of L-glutamate release. AnalChimActa 2009;645(1-2):86-91.
11. Johnson JL, Ellis BA, Munafo DB, et al. Gene transfer and expression in human neutrophils. The phox homology domain of p47phox translocates to the plasma membrane but not to the membrane of mature phagosomes. BMC Immunol 2006;7:28.
12. Dracheva S, Marras SA, Elhakem SL, et al. N-methyl-D-aspartic acid receptor expression in the dorsolateral prefrontal cortex of elderly patients with schizophrenia. Am J Psychiatry 2001;158(9):1400-1410.
